# Exact and Computationally Robust Solutions for Cylindrical Magnets Systems with Programmable Magnetization

**DOI:** 10.1002/advs.202301033

**Published:** 2023-07-17

**Authors:** Federico Masiero, Edoardo Sinibaldi

**Affiliations:** ^1^ Biorobotics Institute Scuola Superiore Sant'Anna viale Rinaldo Piaggio 34 Pontedera 56025 Italy; ^2^ Department of Excellence in Robotics and AI Scuola Superiore Sant'Anna piazza Martiri della Libertà 33 Pisa 56127 Italy; ^3^ Istituto Italiano di Tecnologia via Morego 30 Genoa 16163 Italy

**Keywords:** cylindrical magnets systems, programmable magnetization, exact analytical solution, magnetic field and gradient, magnetic force and torque, magnetic actuation

## Abstract

Magnetic systems based on permanent magnets are receiving growing attention, in particular for micro/millirobotics and biomedical applications. Their design landscape is expanded by the possibility to program magnetization, yet enabling analytical results, crucial for containing computational costs, are lacking. The dipole approximation is systematically used (and often strained), because exact and computationally robust solutions are to be unveiled even for common geometries such as cylindrical magnets, which are ubiquitously used in fundamental research and applications. In this study, exact solutions are disclosed for magnetic field and gradient of a cylindrical magnet with generic uniform magnetization, which can be robustly computed everywhere within and outside the magnet, and directly extend to magnets systems of arbitrary complexity. Based on them, exact and computationally robust solutions are unveiled for force and torque between coaxial magnets. The obtained analytical solutions overstep the dipole approximation, thus filling a long‐standing gap, and offer strong computational gains versus numerical simulations (up to 10^6^, for the considered test‐cases). Moreover, they bridge to a variety of applications, as illustrated through a compact magnets array that could be used to advance state‐of‐the‐art biomedical tools, by creating, based on programmable magnetization patterns, circumferential and helical force traps for magnetoresponsive diagnostic/therapeutic agents.

## Graphical abstract

Exact and computationally robust solutions are disclosed for cylindrical magnets with generic (axial and diametric) uniform magnetization, which overstep the widely used (and often strained) dipole approximation, and enable the design of magnets systems with programmable magnetization. While offering strong computational gains versus numerical simulations, these analytical results bridge to several applications, in particular in the micro/millirobotics and biomedical field.

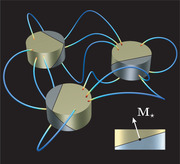

## 1 Introduction

Over the last two decades, magnetic systems and methods for remote actuation and localization have attracted increasing attention, in particular concerning micro/millirobots and miniature devices for biomedical applications, also thanks to the fact that biological tissues are essentially transparent and safely exposed to (relatively slow‐varying and low‐intensity) magnetic fields.^[^ ^1^, ^2^, ^3^, ^4^ ^]^ Progresses in manufacturing are further expanding the design landscape through the possibility to code magnetization profiles in soft magnetoresponsive composites.^[^ ^5^, ^6^, ^7^, ^8^ ^]^ Both current coils systems^[^ ^9^, ^10^ ^]^ and permanent magnets were adopted as field sources. Compared to coils, which permit higher‐frequency modulation and the possibility to switch off the field, permanent magnets can generate stronger fields and gradients, and their application is not restricted by wiring and cooling requirements, thus fostering their attractiveness for heat‐sensitive biomedical procedures.^[^ ^11^ ^]^ As a matter of fact, permanent magnets, and specifically cylindrical magnets in most cases, were used to actuate micro/millirobots and magnetoresponsive agents in air and (biological) fluids/tissues^[^ ^12^, ^13^, ^14^, ^15^, ^16^ ^]^ (by also enabling complementary localization^[^ ^17^, ^18^ ^]^), catheters,^[^ ^19^ ^]^ endoscopes,^[^ ^20^ ^]^ capsules/pills,^[^ ^21^, ^22^ ^]^ building blocks for stable assemblies^[^ ^23^ ^]^ and flexible pumps prospectively functional to soft robotic hearts.^[^ ^24^ ^]^ Moreover, magnetic localization of cylindrical magnets was investigated, for example, for controlling robotic prostheses^[^ ^25^ ^]^ and in wearable sensing systems for rehabilitation.^[^ ^26^ ^]^ Furthermore, (rigid) permanent magnets with simple geometries, such as cylinders and cuboids, were used as external actuation sources for a variety of (deformable) soft magnetoresponsive systems, such as soft magnetic robots^[^ ^27^, ^28^ ^]^ and active substrates for mechanobiology investigations,^[^ ^29^ ^]^ by also enabling shape‐memory and stiffness modulation in composite elastomers.^[^ ^30^ ^]^ Cylindrical magnets, in particular, were further used to actuate soft magnetoresponsive tools for endoluminal navigation,^[^ ^31^, ^32^ ^]^ bellow actuators,^[^ ^8^ ^]^ bioinspired millirobots,^[^ ^33^ ^]^ and substrates for fluid and solid transport.^[^ ^34^ ^]^ Finally, cylindrical (and cuboid) magnets were also exploited for magnetization coding in soft magnetoresponsive materials.^[^ ^35^, ^36^, ^37^ ^]^

In several cases, in order to cope with the complexity of non‐linear magnetic interactions, analytical models for magnets were based on the classical dipole approximation,^[^ ^38^, ^39^ ^]^ both for actuation and localization problems.^[^ ^21^, ^40^ ^]^ Yet it is well known that such an approximation applies for working distances sensibly greater than the characteristic size of the magnetic source, and this condition is often stretched, as occurring, for example, in those biomedical applications where tiny magnetoresponsive objects (devised to operate in anatomical districts) impose relatively cumbersome external magnetic sources (for the magnetic interaction to be effective enough). As a matter of fact, the inadequacy of the dipole approximation was often remarked^[^ ^41^ ^]^ and, in most cases, numerical simulations had to be alternatively performed.^[^ ^8^, ^13^, ^14^, ^15^, ^16^, ^29^, ^34^, ^35^ ^]^ The dipole approximation cannot be applied even for miniature systems where magnets are ingeniously assembled at relatively short distances. Consequently, with reference to cylindrical magnets (and apart from specific expressions only holding on the cylinder axis,^[^ ^42^ ^]^ and approaches combining series‐expansion expressions with numerical calculations^[^ ^43^ ^]^), numerical simulations were exploited even for miniature components/systems,^[^ ^44^ ^]^ including magnetic springs based on coaxial magnets for both linear and rotational mechanisms.^[^ ^45^, ^46^ ^]^

As outlined above, magnets systems were most commonly addressed by means of numerical simulations, which, however, permit to master (and thus leverage) the underlying physical phenomena only in a restricted number of cases and, in general, at higher computational costs. Conversely, analytical approaches immediately contribute to knowledge build‐up (for instance, in terms of scaling laws), and they can offer computationally inexpensive, priceless contributions for conceiving and further developing magnets systems. Yet fundamental solutions, namely complete analytical solutions allowing to exactly and robustly compute both magnetic field and gradient, associated in particular with generic uniform magnetization, are still to be unveiled for many commonest geometries, including cylindrical magnets. This lack is due to the fact that, although the underlying problem formulation is classical,^[^ ^39^ ^]^ related derivations involve non‐trivial functions, including elliptic integrals.^[^ ^47^ ^]^ As a matter of fact, only one exact solution has been recently disclosed for magnetic field and gradient, which is limited to the case of axial magnetization, and which anyway requires two distinct representations in order to circumvent computational singularities.^[^ ^48^ ^]^ Moreover, some solutions for cylindrical magnets with diametric magnetization have been recently reported, whose computation, however, is hampered by singularities,^[^ ^49^, ^50^ ^]^ and which are anyway limited to the magnetic field only.^[^ ^49^, ^50^, ^51^ ^]^ Furthermore, an analytical solution has been recently reported for uniformly magnetized cylindrical tiles, which, however, requires multiple representations to circumvent computational singularities, and which is nonetheless limited to magnetic field only.^[^ ^52^ ^]^ Corresponding gaps in the expanding field of magnetic actuation remain. For instance, exact analytical solutions for force and torque between cylindrical magnets, which are of utmost utility for the design of related systems, are lacking: only the force between coaxial magnets with axial magnetization was disclosed (either by assuming the same radius for both magnets^[^ ^53^ ^]^ or by allowing for different radii^[^ ^54^, ^55^, ^56^ ^]^), whereas analytical expressions for force and torque between coaxial magnets with diametric magnetization have not been achieved so far. This lack of fundamental analytical solutions, in particular for cylindrical magnets with generic magnetization, is reflected by the fact that increasingly ambitious scientific investigations and applications are being tackled mainly based on experimental approaches, possibly complemented by case‐specific and computationally expensive numerical simulations.^[^ ^1^, ^2^, ^28^, ^57^ ^]^ In order to design and develop cylindrical magnets systems with programmable magnetization, exact and computationally robust, complete solutions are therefore strategically sought, for a wide spectrum of applications, and to complement the development of soft magnetoresponsive systems as illustrated above.

This study fills the aforementioned gaps, by providing a manifold original contribution. First, exact analytical solutions are unveiled for both magnetic field and gradient of cylindrical magnets with generic uniform magnetization, thus advancing axial and diametric results within a unifying solution framework. Specifically, complete solutions are disclosed, based on the involved intrinsic entities (namely magnet geometry, pose and magnetization), which can be robustly computed everywhere within and outside the magnets, thus outstripping the dipole approximation. Said solutions only presume a uniform generic magnetization (no additional hypotheses restrict their applicability), and they directly extend, by superposition, to cylindrical magnets systems of arbitrary complexity. Moreover, thanks to the above results, exact and computationally robust analytical solutions are reported for the force and torque between coaxial magnets, including magnets with diametric magnetization, thus also extending the analytical toolkit for magnetic actuation. Specifically, compact expressions are provided, which seamlessly account for generic values of the dimensions of the magnets and their relative distance, thus further overstepping the dipole approximation. Furthermore, all the obtained solutions for magnetic field, gradient, force and torque can be computed by calling a single function, namely the so‐called Bulirsch integral C (Section S1, Supporting Information):

$$\begin{array}{c} C\left(k_{c},p,a,b\right):=\int_{0}^{\pi /2}\frac{a\cos^{2}\psi +b\sin^{2}\psi }{\left(\cos^{2}\psi +p\sin^{2}\psi \right)\sqrt{\cos^{2}\psi +k_{c}^{2}\sin^{2}\psi }}\;d\psi , \end{array}$$

which is commonly available via software libraries.^[^ ^58^ ^]^ In addition, in order to highlight the potential for practical application of the above results, and considering cylindrical magnets arrays proposed for manipulation and biomedical systems,^[^ ^13^, ^15^, ^34^ ^]^ an illustrative application is concisely introduced, based on a compact array allowing to create both circumferential and helical force traps for magnetoresponsive agents, thanks to programmable magnetization patterns. Specifically, superparamagnetic agents are considered, because they are increasingly proposed for both diagnostic and therapeutic approaches (such as magnetic resonance imaging, drug delivery and thermotherapy).^[^ ^59^ ^]^ Finally, derived computational gains are also shown, through comparisons with (finite element) numerical simulations, and self‐contained implementations of the obtained solutions are provided, for the benefit of scientists and potential users from a broader readership.

## 2 Results

**Figure 1:**
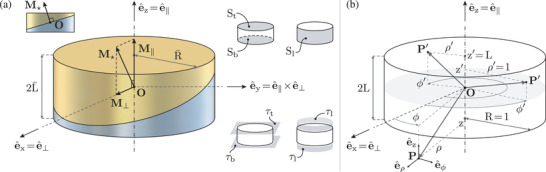
Cylindrical magnet with generic uniform magnetization. a) Magnet size (radius $\bar{R}$, half‐height $\bar{L}$), pose (origin **O**, axial direction ${\hat{e}}_{∥}$), and magnetization (**M**_⋆_). Magnetization, in particular, is decomposed into axial (**M**_∥_) and diametric (**M**_⊥_) contributions: **M**_⋆_ = **M**_∥_ + **M**_⊥_. Gold/silver colors consistently remind of magnetic poles. b) Non‐dimensional cylindrical coordinates: illustration for two generic points **P**′ on the surface of the magnet, and a generic point **P** outside the magnet. The unit vectors systems $\left\{{\hat{e}}_{\rho },{\hat{e}}_{\phi },{\hat{e}}_{z}\right\}$ and $\left\{{\hat{e}}_{⊥},{\hat{e}}_{∥}\;\times \;{\hat{e}}_{⊥},{\hat{e}}_{∥}\right\}$ represent the considered cylindrical and intrinsic (Cartesian) frames, respectively. We obtained exact and computationally robust analytical solutions, for both magnetic field and field gradient, at generic points **P** outside or inside the magnet (and, by superposition, our solutions extend to cylindrical magnets systems of arbitrary complexity, in terms of both spatial arrangement and magnetization pattern).

**Figure** **1** introduces a cylindrical magnet with generic uniform magnetization. Figure 1a shows the involved intrinsic entities: magnet size (defined by radius $\bar{R}$ and half‐height $\bar{L}$), pose (given by origin **O** and axial direction ${\hat{e}}_{∥}$, bold symbols and $\hat{\cdot }$ hereafter denoting vectors and unit vectors, respectively), and magnetization **M**_⋆_ = **M**_∥_ + **M**_⊥_ (decomposed into axial $M_{∥}\;=\;M_{∥}\;{\hat{e}}_{∥}$ and diametric $M_{⊥}\;=\;M_{⊥}\;{\hat{e}}_{⊥}$ contributions). Gold/silver colors remind (in all figures) of magnetic poles consistent with **M**_⋆_. Moreover, the adopted cylindrical and (Cartesian) intrinsic frames are shown, respectively denoted by $\left\{{\hat{e}}_{\rho },{\hat{e}}_{\phi },{\hat{e}}_{z}\right\}$ and $\left\{{\hat{e}}_{⊥},{\hat{e}}_{∥}\;\times \;{\hat{e}}_{⊥},{\hat{e}}_{∥}\right\}$, as well as the non‐dimensional coordinates used to preserve physical consistency during the derivation (based on $\bar{R}$ as reference length, Figure 1b). With reference to the figure at hand, it is worth anticipating that we obtained exact and computationally robust analytical solutions, for both magnetic field (**H**) and field gradient (grad(**H**)), at generic points **P** either outside or inside the magnet.

**Table** **1** shows the solution for the magnetic field **H** = **H**_∥_ + **H**_⊥_. Cylindrical components for axial and diametric contributions are reported through Equations (1), (2) and (3), (4), (5), respectively, while Equation 6 compactly provides the solution in purely vectorial terms. **Table** **2** shows the solution for the field gradient grad(**H**) = grad(**H**_∥_) + grad(**H**_⊥_), in terms of its matrix representations *G* ^(*cyl*)^ and *G* in the cylindrical and intrinsic frame, respectively. Axial and diametric contributions for *G* ^(*cyl*)^ are reported through Equations (7), (8), (9) and (10), (11), (12), (13), (14), respectively, while Equation 15 compactly provides *G*. All the exact solutions in Tables 1 and 2 can be computed by solely calling C; self‐contained implementation details (including auxiliary expressions for *c*_ϕ_, *s*_ϕ_, *f*_0_‐*f*_5_, **u**, **v**, ${\tilde{J}}_{∥}$ and ${\tilde{J}}_{⊥}$) are reported in Section 4 for the benefit of generic readers keen on directly using the obtained solutions.

**Table 1:** Exact solution for the magnetic field

Components (cylindrical frame)	Complete solution (frameless)
(1) $\;H_{∥\rho }\;\pi =M_{∥}\;\left(-\rho \;f_{3}\right)$ (2) $\;H_{∥z}\;\pi =M_{∥}\;\left(\;f_{0}+2\;f_{1}\right)$ (3) $\;H_{⊥\rho }\;\pi =M_{⊥}\;c_{\phi }\;\left(\rho \;f_{2}-f_{1}\right)$ (4) $\;H_{⊥\phi }\;\pi =M_{⊥}\;s_{\phi }\;\left(\rho \;f_{2}+f_{1}\right)$ (5) $\;H_{⊥z}\;\pi =M_{⊥}\;c_{\phi }\;\left(-\rho \;f_{3}\right)$	(6) $\;H=\left(\;f_{0}\;M_{∥}\;+f_{1}\left(2\;M_{∥}-M_{⊥}\right)+f_{2}\;u+f_{3}\;v\right)\;/\;\pi$

**Table 2:** Exact solution for the magnetic field gradient

Components (*G* ^(*cyl*)^, cylindrical frame)	Complete solution (*G*, intrinsic frame)
(7) $\;{\bar{\rho }}^{-1}\;\left(\partial_{\phi }H_{∥\phi }+H_{∥\rho }\right)\;\pi \;\bar{R}\;=M_{∥}\;\left(-f_{3}\right)$ (8) $\;\partial_{\bar{\rho }}H_{∥z}\;\pi \;\bar{R}\;=M_{∥}\;\left(\;f_{4}\right)$ (9) $\;\partial_{\bar{z}}H_{∥z}\;\pi \;\bar{R}\;=M_{∥}\;\left(\;f_{5}\right)$ (10) $\;{\bar{\rho }}^{-1}\;\left(\partial_{\phi }H_{⊥\rho }-H_{⊥\phi }\right)\;\pi \;\bar{R}\;=M_{⊥}\;s_{\phi }\;\left(-2\;f_{2}\right)$ (11) $\;{\bar{\rho }}^{-1}\;\left(\partial_{\phi }H_{⊥\phi }+H_{⊥\rho }\right)\;\pi \;\bar{R}\;=M_{⊥}\;c_{\phi }\;\left(2\;f_{2}\right)$ (12) $\;\partial_{\bar{\rho }}H_{⊥z}\;\pi \;\bar{R}\;=M_{⊥}\;c_{\phi }\;\left(\;f_{3}-f_{5}\right)$ (13) $\;{\bar{\rho }}^{-1}\;\left(\partial_{\phi }H_{⊥z}\right)\;\pi \;\bar{R}\;=M_{⊥}\;s_{\phi }\;\left(\;f_{3}\right)$ (14) $\;\partial_{\bar{z}}H_{⊥z}\;\pi \;\bar{R}\;=M_{⊥}\;c_{\phi }\;\left(\;f_{4}\right)$	(15) $G=\left(M_{∥}\;{\tilde{J}}_{∥}+\;M_{⊥}\;{\tilde{J}}_{⊥}\right)\;/\;\left(\pi \bar{R}\right)$

It is to be remarked that Equations 6 and 15, which also circumvent the indeterminacy of the cylindrical components associated with ${\hat{e}}_{\rho }$ and ${\hat{e}}_{\phi }$ on the cylinder axis, provide the complete solutions, in terms of the involved intrinsic entities. The cylindrical components, however, permit to easily see that, while **H** is continuous both outside and inside the magnet, H_⊥ρ_ is discontinuous across the lateral surface of the magnet (S_l_ in Figure 1a), whereas H_∥z_ is discontinuous across the bottom and top surfaces (S_b_ and S_t_, respectively). Such discontinuities are consistent with the well‐known “jump” condition on the magnetic induction **B**, namely $\hat{n}\;\cdot \;⟦B⟧\;=\;0$ ^[^ ^39^ ^]^ (where ⟦·⟧ denotes the difference between outside and inside limit values: outside minus inside, let us say); indeed, $⟦H_{⊥\rho }⟧\;=\;M_{⊥}\cos\phi$ across S_l_, and $⟦H_{∥z}⟧\;=\;M_{∥}$ across S_b_ and S_t_. Finally, the exact solutions in Tables 1 and 2, and in particular the complete solutions in Equations 6 and 15, can be robustly evaluated (no singularities arise) in the whole computational domain, thus providing analytical results that inherently outperform the dipole approximation.

**Figure 2:**
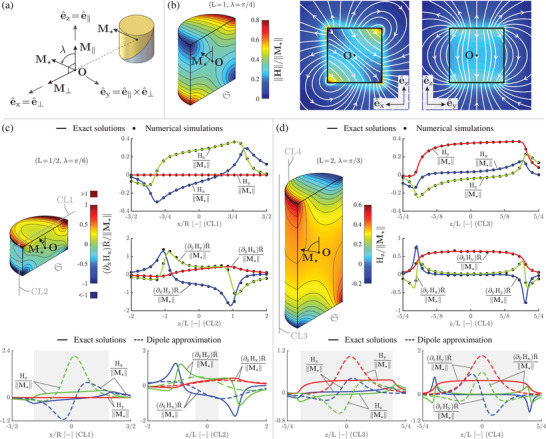
Magnetic field and gradient of a single magnet: illustrative results. a) Parameterization (schematic) of magnetization orientation through the angle λ. b) Illustrative results for (L = 1, λ = π/4): contour plot of ||**H**|| on S, and **H** field lines on the ${\hat{e}}_{x}$‐${\hat{e}}_{z}$ and ${\hat{e}}_{y}$‐${\hat{e}}_{z}$ planes. The field lines (white curves over ||**H**|| intensity plots) highlight physically‐consistent **H** discontinuities across the surface of the magnet. c) Illustrative results for (L = 1/2, λ = π/6): contour plot of $\partial_{\bar{x}}H_{x}$ on S, and comparison between the exact solutions and the results provided by numerical (finite element) simulations and by the dipole approximation. d) Illustrative results for (L = 2, λ = π/3): contour plot of H_z_ on S, and comparison between the exact solutions and the results provided by numerical simulations and by the dipole approximation. In c,d), selected field and gradient components are shown, on chosen cut‐lines (CL1‐CL4). The exact analytical solutions provide the same results as the numerical simulations (considering the whole domain, the relative difference was below 10^−2^ and 2 · 10^−2^ for **H** and grad(**H**), respectively, in the whole λ range and for each component), yet with strong computational gains (above 10^3^). The dipole approximation introduces remarkable errors (even outside the gray‐shadowed intervals, within which the approximation accuracy is expected to deteriorate), thus further underlining the value of the exact solutions.

**Figure** **2** shows illustrative results for **H** and grad(**H**) of a single magnet. Specifically, Figure 2a shows the angle λ introduced to parameterize the generic magnetization orientation, while Figure 2b–d show illustrative contour plots of **H** and grad(**H**), for selected values of L and λ, on a chosen surface S close to the magnet (where our solutions effortlessly apply, whereas the dipole approximation is challenged). Moreover, field lines in Figure 2b highlight physically‐consistent **H** discontinuities across the magnet surface (only due to **M**_∥_ on the ${\hat{e}}_{y}$‐${\hat{e}}_{z}$ plane, even to **M**_⊥_ on the ${\hat{e}}_{x}$‐${\hat{e}}_{z}$ plane), while Figure 2c,d also compare the obtained exact analytical results with those provided by numerical (finite element) simulations, and by the dipole approximation. In particular, the agreement between exact solutions and numerical simulations is shown, for chosen **H** and grad(**H**) components, on selected cut‐lines (CL1‐CL4). Beyond these specific illustrations, the relative difference was below 10^−2^ and 2 · 10^−2^ for **H** and grad(**H**), respectively, over the whole λ range (with slightly larger differences for the gradient mainly due to numerical differentiation within the finite element solver). Yet computational times were remarkably different: *O*(10^2^)‐*O*(10^3^) s (setup and postprocessing times excluded) to run a numerical simulation, versus *O*(10^−5^)‐*O*(10^−4^) s (without algorithmic optimization) to compute **H** and grad(**H**) at a point via the analytical results. Detailed quantification of speed‐up factors was clearly beyond the present scope; however, assuming to compute field and gradient at *O*(10^3^) points, the exact solutions offer strong computational gains (above 10^3^) compared to numerical simulations. Finally, the dipole approximation introduces remarkable errors (even outside the gray‐shadowed intervals, within which the approximation accuracy is expected to deteriorate because the cut‐lines intersect the smallest sphere bounding the magnet), thus further underlining the value of the exact solutions achieved in this study.

**Figure 3:**
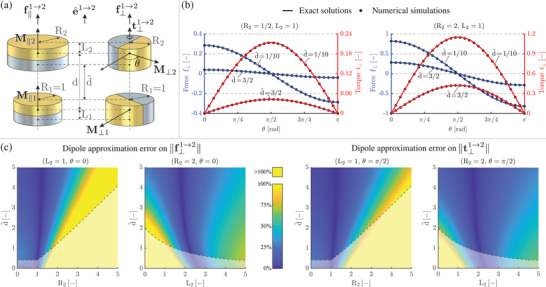
Magnetic force and torque between coaxial magnets: illustrative results. a) Coaxial magnets (schematic): with axial magnetizations (left); with diametric magnetizations at relative angular shift θ (right). b) Normalized force f_⊥_ and torque t_⊥_ between magnets with diametric magnetization: comparison between the exact solutions and the results provided by numerical (finite element) simulations, for (R_2_ = 1/2, L_2_ = 1) and (R_2_ = 2, L_2_ = 1), and selected values of the face‐to‐face distance $\tilde{d}$. The exact analytical solutions provide the same results as the numerical simulations (the relative difference was below 10^−2^ for both force and torque, in the whole θ range), yet with strong computational gains (well above 10^3^). c) Relative error (with respect to the exact solutions) introduced when computing $\left|\right|f_{⊥}^{1\to 2}\left|\right|$ and $\left|\right|t_{⊥}^{1\to 2}\left|\right|$ via the dipole approximation, by varying $\tilde{d}$ and R_2_ (resp. L_2_) once fixed L_2_ = 1 (resp. R_2_ = 2). Considerable discrepancies appear (even outside the regions defined by a transparent white layer, within which the dipole approximation accuracy is expected to deteriorate), thus further remarking the merit of the exact solutions (grounded on those previously obtained for field and gradient).

The explicit solutions obtained for **H** and grad(**H**) opened up the possibility to achieve further exact results, and we seized this opportunity to tackle related gaps in magnetic actuation. Specifically, we addressed the interactions between two coaxial cylindrical magnets C1 (radius ${\bar{R}}_{1}$, half‐height ${\bar{L}}_{1}$) and C2 (radius ${\bar{R}}_{2}$, half‐height ${\bar{L}}_{2}$), at relative distance $\bar{d}\;\geq \;{\bar{L}}_{1}\;+\;{\bar{L}}_{2}$ along direction ${\hat{e}}^{1\to 2}$, as shown in **Figure** **3**a (non‐dimensional schematic, based on ${\bar{R}}_{1}$ as reference length). By assuming axial magnetizations **M**_∥1_ and **M**_∥2_, we first computed the corresponding force $f_{∥}^{1\to 2}$ exerted by C1 on C2 (torque being null, by symmetry); by assuming diametric magnetizations **M**_⊥1_ and **M**_⊥2_ relatively shifted by an angle θ, we then computed the related force $f_{⊥}^{1\to 2}$ and torque $t_{⊥}^{1\to 2}$. The achieved exact solutions are reported in **Table** **3**, where μ_0_ denotes vacuum magnetic permeability. All the exact solutions in Table 3 can be computed by solely calling C; self‐contained implementation details (including auxiliary expressions for η_*f*_ and ζ_*t*_) are reported in Section 4, ready for use by any interested readers. Moreover, they can be robustly computed for generic magnets size and relative distance, down to the limit case of magnets in contact with each other, thus providing analytical results that inherently outperform those achievable through the dipole approximation. Figure 3b illustrates the agreement between exact analytical solutions and numerical (finite element) simulations, for selected values of R_2_ and L_2_, considering the normalized force f_⊥_ and torque t_⊥_ associated with diametric magnetizations. The relative difference was below 10^−2^ for both force and torque, in the whole θ range, with strong computational gains (well above 10^3^) for the exact solutions (gains are higher than those for field/gradient, since force/torque are scalar values resulting from a spatial integration that is already accounted for by the exact analytical solutions). Furthermore, Figure 3c illustrates the relative error (with respect to the exact solutions) introduced when computing $\left|\right|f_{⊥}^{1\to 2}\left|\right|$ and $\left|\right|t_{⊥}^{1\to 2}\left|\right|$ by means of the dipole approximation, by varying the face‐to‐face distance $\tilde{d}$ and R_2_ (resp. L_2_) for selected values of L_2_ (resp. R_2_), once fixed θ so as to maximize force and torque intensity (consistently with Figure 3b), for ease of presentation. The dipole approximation introduces considerable discrepancies (even outside the regions defined by a transparent white layer, within which the approximation accuracy is expected to deteriorate because at least one portion of a magnet intersects the smallest sphere bounding the other one), thus further remarking the merit of the exact solutions. Let us observe that the relative errors on $\left|\right|f_{⊥}^{1\to 2}\left|\right|$ are also representative of those associated with $\left|\right|f_{∥}^{1\to 2}\left|\right|$, because both forces share the same “shape function” η_*f*_ in Table 3.

**Table 3:** Exact solutions for the force and torque between coaxial magnets

Axial magnetization	Diametric magnetization
(16) $\;f_{∥}^{1\to 2}=-\mu_{0}\;{\bar{R}}_{1}\;{\bar{R}}_{2}\;\;\eta_{f}\left(R_{2},L_{1},L_{2},d\right)\;\;\left(M_{∥1}\;\cdot \;M_{∥2}\right)\;{\hat{e}}^{1\to 2}$	(17) $f_{⊥}^{1\to 2}=\frac{\mu_{0}}{2}\;{\bar{R}}_{1}\;{\bar{R}}_{2}\;\;\eta_{f}\left(R_{2},L_{1},L_{2},d\right)\;\;\left(M_{⊥1}\;\cdot \;M_{⊥2}\right)\;{\hat{e}}^{1\to 2}$ (18) $t_{⊥}^{1\to 2}=\frac{\mu_{0}}{6}\;{\bar{R}}_{1}^{2}\;{\bar{R}}_{2}\;\;\zeta_{t}\left(R_{2},L_{1},L_{2},d\right)\;\;\left(M_{⊥1}\;\times \;M_{⊥2}\right)$

**Figure 4:**
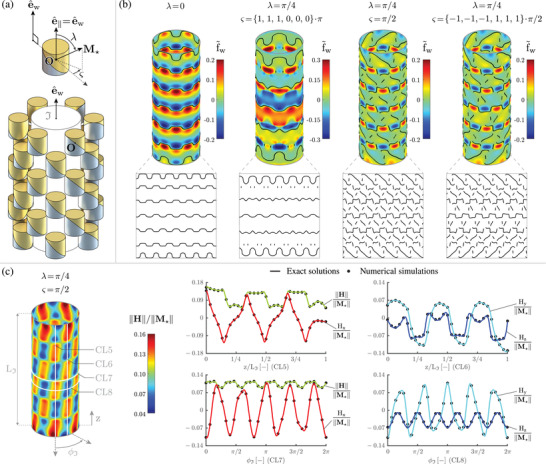
Magnetization patterning on a compact cylindrical magnets array. a) Schematic of a 6‐ring array with 6 magnets per ring, also showing the cylindrical workspace I and the angles (λ, ς) used to individually parameterize magnetization orientation. b) Illustrative results for selected magnetization patterns: intensity plots of the normalized axial force ${\tilde{f}}_{w}$ acting on superparamagnetic agents (modeled as point dipoles) located on the workspace lateral surface, and associated magnetic traps (superimposed black curves, highlighted through insets that unroll the considered lateral surface). Compared to the circumferential traps created with purely axial magnetizations (λ = 0), more elaborate schemes also involving helical traps with spatially varied chirality can be obtained by varying ς from ring to ring, while keeping λ = π/4 for all the magnets, for simplicity. Given a compact array configuration, this possibility to modulate the force traps by re‐programming the magnetization pattern (here rotating the magnets around their common axis) could be used, for example, to advance endoluminal tools for magnetic retrieval of diagnostic/therapeutic agents in complex/swirling biological flows. Beyond this illustration, richer design spaces can be inexpensively explored thanks to the obtained exact solutions. c) Illustrative results for the (λ = π/4, ς = π/2) magnetization pattern: contour plot of ||**H**|| on the workspace lateral surface, and comparison between the exact solutions and the results provided by numerical (finite element) simulations, through selected field components on chosen cut‐lines (CL5‐CL8, white‐highlighted over the aforementioned contour). The exact analytical solutions provide the same results as the numerical simulations (the relative difference was below 2 · 10^−2^, smaller differences being only hampered by the physical memory required by numerical simulations), yet with very strong computational gains (around 10^6^). This test‐case, beyond its specificity, sheds further light on the advantages enabled by the exact solutions determined in this study.

Finally, in order to illustrate the effective usability of the achieved analytical results closer to real‐world systems, and considering state‐of‐the‐art actuation arrays aimed to trap magnetoresponsive agents in biological fluids/tissues,^[^ ^13^, ^15^ ^]^ we then addressed a cylindrical magnets array. Specifically, we considered the system sketched in **Figure** **4**a, composed of 6 rings, each with 6 magnets aligned with the axial direction ${\hat{e}}_{w}$ of the cylindrical workspace I, and devised to create force traps for superparamagnetic agents (modeled as point dipoles) located on the workspace lateral surface (superparamagnetic particles, indeed, are increasingly proposed for both diagnostic and therapeutic applications,^[^ ^2^, ^28^, ^59^ ^]^ including magnetic targeting/retrieval in/from biological flows^[^ ^12^, ^13^ ^]^). By varying **M**_⋆_ (parameterized by the angles λ and ς for each magnet), we aimed to illustrate the possibility to program the magnetization pattern so as to create, based on the same compact array configuration, both circumferential and helical magnetic traps, as potentially favorable, for example, for retrieval in complex/swirling flows (which is still to be demonstrated in literature). It should be noticed that the possibility to exactly and inexpensively compute such traps even close to the magnets was specifically enabled by the solutions obtained for **H** and grad(**H**) in this study. Figure 4b illustrates the resulting normalized axial force ${\tilde{f}}_{w}$, together with the corresponding magnetic traps (black curves over ${\tilde{f}}_{w}$ intensity plots, further highlighted through corresponding insets). Starting from the “base” configuration with purely axial magnetizations (λ = 0) for which circumferential traps can be intuitively created (and traps wiggling could be reduced by increasing magnets density), the other three test‐cases show the possibility to modulate the traps by simply varying ς from ring to ring (while keeping λ = π/4 for all the magnets, for simplicity). In particular, circumferential trapping stripes can be created by keeping each **M**_⋆_ in the corresponding sagittal plane (namely the plane passing through the workspace axis and the corresponding magnet center **O**), for example, with ς = {1, 1, 1, 0, 0, 0} · π, whereas helical traps can be introduced with magnetizations perpendicular to the corresponding sagittal planes, for example, with ς = π/2 or ς = {−1, −1, −1, 1, 1, 1} · π/2. The latter two test‐cases also highlight the possibility to locally modulate traps chirality, which could be functional to specific tools/applications. This illustration, necessarily simplified and concise to keep the study focused, underlines the concrete opportunity to leverage our exact solutions in order to inexpensively explore richer design spaces, as discussed in Section 3. Figure 4c further illustrates the (λ = π/4, ς = π/2) magnetization pattern, through the contour plot of ||**H**|| on the workspace lateral surface, and by showing the agreement between exact solutions (obtained by superposition of those individually associated with each magnet in the array) and numerical (finite element) simulations, on selected cut‐lines (CL5‐CL8, white‐highlighted over the aforementioned contour). The relative difference was below 2 · 10^−2^, with smaller differences only hampered by memory constraints. Indeed, the considered finite element run required a peak memory very close to the total physical memory of the used computer, because of the relatively high number of elements needed to suitably resolve the magnetic field variation close to the surface of the magnets (and in particular to their edges), also considering their arrangement. Consistently, computational times were strikingly different: the numerical simulation took *O*(10^4^) s (setup and postprocessing times excluded), while the exact solutions were computed in *O*(10^−2^) s (without algorithmic optimization), thus offering a very strong computational gain (about 10^6^). Beyond its specificity, the considered test‐case sheds further light on the advantages enabled by the results of the present study, as further discussed in Section 3.

## 3 Discussion and Conclusions

The number of studies involving magnetic methods/systems, in particular for micro/millirobotics and biomedical applications, has been strongly increasing in recent years. Novel functionalities and approaches are being enabled by the integration of (rigid) magnets into systems with programmable magnetization patterns, and by their use for developing (deformable) magnetoresponsive soft‐material systems, in particular as external actuation sources and for magnetization coding.^[^ ^1^, ^4^, ^28^ ^]^ Yet technological and implementation advances, possibly supported by numerical simulations, seem not to be correspondingly backed by progresses in analytical tools, which are crucial to develop magnetic systems. Indeed, the physics knowledge‐based approach cannot be replaced, in general, with case‐specific numerical simulations, whose computational costs, moreover, can soon become unsustainable as the complexity of the systems increases. At the same time, the dipole approximation is commonly used also where its applicability can be questioned (as, for instance, when modeling relatively cumbersome magnetic sources devised to actuate biomedical devices in anatomical districts). Even past efforts taken to identify permanent magnet geometries whose field could be optimally fit by the dipole approximation^[^ ^60^ ^]^ underline the impactful constraint posed by the lack of more descriptive analytical models. The fact is that analytical solutions allowing to determine magnetic fields and gradients, exactly and robustly in the whole computational domain, are still to be unveiled for many commonest geometries, including cylindrical magnets that are almost ubiquitously used both in fundamental research and applications.

Challenged by the above striking and long‐standing theoretical gap, and considering the potential impact of the sought solutions on a gamut of research scopes and practical applications, we tackled the fundamental scientific problem of exactly and robustly computing both magnetic field and field gradient for cylindrical magnets with generic (axial and diametric) uniform magnetization (Figure 1). This study unveils the complete solutions through Tables 1 and 2, and in particular via Equations 6 and 15 that determine magnetic field and gradient, respectively, through a single compact expression. These solutions, which hold both within and outside the magnets (while also describing physically‐consistent discontinuities across their surface, Figure 2b), are exact, and they can be robustly computed in the whole domain, thus intrinsically overstepping the dipole approximation (Figure 2c,d). Moreover, the obtained solutions extend, by superposition, to hollow cylindrical magnets and cylindrical magnets systems of arbitrary complexity, in terms of both spatial arrangement and magnetization patterns.

The solutions determined in the present study surpass the related analytical results reported in literature, for the following multiple aspects. (i) Our analytical framework provides exact solutions for both magnetic field and gradient, thus outstripping ref. [48], which is limited to axial magnetization, and refs. [49, 50, 51, 52], which did not determine the gradient solution. Indeed, no analytical solutions were previously achieved for the gradient when considering diametric magnetization. (ii) Our solutions (including the cylindrical components that specify axial and diametric contributions for magnetic field and gradient) can be robustly computed in the whole computational domain. In particular, they do not suffer from singularities on the prolongation τ_l_ of the lateral surface of the magnet (Figure 1a), where ref. [49] leaves singular expressions and ref. [48] has to introduce an additional representation for computing the solution. (iii) Both for field and gradient, our complete solution is provided by a single expression explicitly accounting for the involved intrinsic entities (namely magnet geometry, pose and magnetization), and such an expression is not limited, in particular, by the representation in cylindrical coordinates (which are not fully defined on the cylinder axis). Specifically, the field solution in Equation 6 is purely vectorial (i.e., it is frameless), and the gradient solution in Equation 15 is expressed as a matrix in the magnet intrinsic frame, which is always well‐defined based on pose and magnetization. (We deliberately pursued this matrix representation, because it can be directly exploited for generic implementations by a wide range of scientists/engineers/users, not necessarily acquainted with more involved mathematical formalism.) Consequently, our field solution does not suffer from singularities on the axis of the magnet, differently from ref. [50]. Moreover, the obtained vectorial solution provides a compact and physically descriptive representation in the whole domain, differently from ref. [51] that leaves both axial and diametric contributions individually expressed through cylindrical components (and reports some Cartesian expressions limited to the axis). The advantage of our solution can be further appreciated by comparing Equation 6 with the considerable number of corresponding expressions reported through multiple tables in ref. [52] where the use of a computer algebra system (to automatically integrate the relevant governing equations) implied to cope with singularities (potentially introduced through the involved integration constants) by considering multiple cases. (iv) Our complete solutions, for both field and gradient, can be computed by calling a single function, namely C. Although quantitative comparative claims on computational cost could be rigorously introduced only based on a purposely‐focused study beyond the present scope (also considering, for instance, hardware/software benchmarks and optimized implementations), some arguments can be drafted in view of the involved functions. In particular, considering that complete elliptic integrals can be compactly computed through C, the computational cost of our complete solutions is expected not to exceed that one of the partial solutions in refs. [48, 49]. Moreover, our complete solutions are expected to be computationally cheaper than the partial solutions in refs. [50, 51, 52], because the latter also involve incomplete elliptic integrals that are more computationally demanding.^[^ ^47^ ^]^ (v) Our analytical framework also enables original solutions for force and torque, which surpass, per se, related analytical results in literature. Specifically, the exact and computationally robust solutions in Table 3, which can be computed by solely calling C, hold for generic magnets size and relative distance, down to the limit case of magnets in contact with each other, thus further outstripping the dipole approximation (Figure 3c). Moreover, besides encompassing previous results on coaxial magnets with axial magnetization,^[^ ^53^, ^54^, ^55^, ^56^ ^]^ our solutions for force and torque between coaxial magnets with diametric magnetization are the first to be reported. To conclude, the above points highlight a manifold substantial advancement compared to previous analytical results.

The scope of our theoretical results is confined by the assumption of uniform magnetization. This limitation is in common with all the previous analytical results recalled in the above paragraph, because, in practice, assuming uniform magnetization is key for enabling analytical treatment and, notwithstanding the inherent simplification compared to real‐world systems, the derived results contributed to build‐up fundamental knowledge on magnetic systems, besides fostering complementary methods (including numerical approaches), experiments design, and implementations. The fact is that the intrinsic non‐linearity of magnetostatics problems does not permit to obtain analytical solutions for fields/gradients associated with generic non‐uniform magnetizations, and this complication is exacerbated when pursuing force/torque solutions, due to additional mathematical difficulties associated with spatial derivation/integration.^[^ ^39^ ^]^ In this regard, it should be noticed that assuming uniform magnetization does not remove, per se, the base challenges, as also reflected, for example, by recent solutions for the field (field only, no gradient) of uniformly magnetized cylindrical tiles, which are not fully analytical and whose computation is hampered by singularities.^[^ ^61^ ^]^ Hence, even by assuming uniform magnetization, obtaining complete analytical solutions, as those disclosed by the present study, remains scientifically challenging and relatively uncommon, at large. At the same time, the assumption of uniform magnetization does not necessarily introduce a detrimental limitation, because rare‐earth magnetic materials nowadays in widespread use, such as Neodymium‐Iron‐Boron (NdFeB), exhibit an almost ideal hard‐magnetic behavior,^[^ ^52^ ^]^ and related real magnets systems can be accurately modeled by assuming uniform magnetization.^[^ ^53^ ^]^ For completeness, we observe that some analytical results were obtained even for hollow cylindrical magnets with ideally circumferential^[^ ^62^ ^]^ or radial magnetization.^[^ ^63^ ^]^ Such solutions (not fully analytical in the latter case), are nonetheless limited to magnetic field components, expressed in particular in cylindrical coordinates, and their application to real magnets could be hampered by the challenge to accurately code a circumferential/radial magnetization profile during manufacturing. That said, our solutions cannot be applied for materials featuring hysteretic magnetic behavior, as well as soft‐magnetic materials (introducing further non‐linear effects) and hard‐magnetic materials with appreciably non‐uniform magnetization (also due, for instance, to shape anisotropy^[^ ^2^, ^28^ ^]^). For those cases, numerical approaches must be pursued, including, for example, moments methods devised to compute demagnetization effects in non‐uniformly magnetized bodies,^[^ ^64^, ^65^, ^66^ ^]^ thus entering a research field beyond the scope of our study. At the same time, the present considerations spur to further leverage our analytical framework in search for additional exact solutions, in particular associated with specific magnetization patterns, for both field/gradient and force/torque. As for the latter, we conclude this paragraph by remarking that the obtained solutions are further limited to coaxial magnets. Indeed, exact force/torque solutions for cylindrical magnets with generic relative pose are, overall, hardly viable because of additional mathematical difficulties (for instance, associated with spatial integration over generic cylindrical domains represented in a common frame). Arguably, this is the reason why, already for non‐coaxial parallel magnets, and by accepting the additional restriction to axial magnetization, no fully analytical solutions have been achieved so far.^[^ ^53^, ^56^ ^]^ The possibility of overcoming, even partially, the above challenges, through an extension of the proposed analytical framework, will be assessed through subsequent investigations.

For the sake of clarity, we also remark that the obtained exact solutions hold for rigid cylindrical magnets: they do not apply to deformable cylindrical magnets, for example, made of soft magnetoresponsive composites. Indeed, magnetoresponsive continua must be modeled by considering that a magnetic field induces body deformations that, in turn, affect the field itself, and this magneto‐mechanical coupling adds to non‐linear material effects (such as, for instance, the hyperelastic behavior of common polymeric matrices used in composites^[^ ^28^ ^]^). Consequently, the possibility to obtain analytical solutions for magnetoelastic problems is further confined to assumed elementary deformations and magnetic fields,^[^ ^67^ ^]^ whereas case‐specific numerical simulations are possibly used to support the development of more realistic systems. There is, however, a concrete connection between our findings and magnetoresponsive continua. In many cases, indeed, the external magnetic sources used to actuate magnetoresponsive soft‐material systems are rigid magnets with uniform magnetization and simple geometries, such as cylinders and cuboids. More in detail, such external magnets are generally displaced/rotated, in a controlled way, for inducing sought deformations/effects on the continuum, as occurring, for example, in ref. [29], where couples of opposite magnets were alternatively displaced along predefined directions for stretching a continuum sample, or in ref. [27], where the considered magnetic slime was deformed in a functional way by moving multiple permanent magnets. In numerical simulations, such pose variations of the external rigid magnets increase computational complexity/costs (either for multiple simulations or for re‐meshing in a single run), on top of the efforts necessary to model the continuum sample. Differently, our exact solutions could be called at runtime in order to inexpensively compute both field and gradient for the external cylindrical sources, as functional to define body forces and torques acting on the continuum. (This argument adds to the advantages offered by our analytical solutions versus numerical simulations, as further discussed below.) In the same spirit, considering the use of cylindrical magnets for modulating the stiffness or the rheological properties of soft magnetoresponsive composites, our results could also be used to quantitatively investigate/characterize magnetorheological elastomers. Similarly, our results could support the design of magnetization profiles to be coded, through external cylindrical magnets, in soft magnetoresponsive composites (by taking advantage of the exact determination of fringe effects around the magnets). In this regard, our solutions could complement recent approaches for the design of magnetization coding, for example, based on a combination of model‐based and data‐driven strategies.^[^ ^36^, ^68^ ^]^ Even in this case, however, subsequent specific investigations are necessary to back stronger and more detailed claims.

Notwithstanding the above discussed limitations, our theoretical solutions offer advantages even from an application point of view. First, being exact, they remove the inherent inaccuracy introduced by the dipole approximation when designing actuation and localization systems. As for the former, they could be used, for example, to accurately model the cylindrical magnets used for manipulating miniature biomedical devices, thus possibly extending ref. [21]. As for the latter, they could be used, for example, for accurately detecting magnets relatively close to sensors in robotic prostheses, thus possibly extending ref. [25]. Moreover, exactly knowing the gradient is profitable for both computations and system design/control. Indeed, it avoids finite‐precision differentiation in numerical solvers, with benefits for robustness/accuracy, as quickly mentioned when comparing exact solutions and numerical simulations for a single magnet (Figure 2c,d). In addition, exactly knowing the gradient fosters system design/control by means of Jacobian‐based optimization methods.^[^ ^20^ ^]^ Furthermore, our solutions in Equation 6 and 15 explicitly account for the key design parameters (i.e., magnets geometry, pose and magnetization) and, given their vectorial character, the designer can trivially exploit superposition (while also taking advantage, to some extent, of physical intuition) in order to explore/define the design space. At the same time, algorithmic design is fostered by the fact that our solutions can be computed by calling a single function. For the sake of illustration, let us consider, for example, the magnetic actuation system reported in ref. [43], based on multiple cylindrical magnets with axial magnetization. Its design was based on a series‐expansion solution in cylindrical coordinates for the magnetic field of each magnet, combined with corresponding frame transformations aimed to represent all the involved contributions in a common reference frame. We respectfully observe that a corresponding design strategy based on our exact field solution would remove the inaccuracies inherently associated with series truncation (as well as the partial indeterminacy of cylindrical coordinates on the axis of each magnet), while also reducing computational complexity, thanks to the use of a single function and the removal of coordinate transformations. In addition, our exact gradient solution could be used to further optimize design (by also considering, for instance, extended targets/constraints), and the advantage of computationally inexpensive vectorial solutions is expected to become more compelling as the number of involved magnets increases. Furthermore, taking advantage of the fact that our solutions were expressly determined for magnets systems with programmable magnetization, we can explore richer design spaces by leveraging magnetization patterning, yet this aspect is further discussed in the following paragraph, for ease of presentation. Moreover, our exact and singularity‐free solutions could be used to also improve the robustness of model‐based localization systems, because sampling a model close to singular points/domains can affect the convergence of the underlying algorithms without clear hints of the almost‐hit singularity, since its detection can be blurred by numerical round‐off, both in simulations and in real‐world embedded processors. In addition, they could be used to increase the number of sensed degrees‐of‐freedom (for instance, in wearable rehabilitation systems^[^ ^26^ ^]^), without sacrificing accuracy. Finally, even the derived exact solutions for force/torque could be used for the design of linear/rotational magnetic springs, including hollow magnets (through clear extensions by superposition), thus replacing case‐specific numerical simulations^[^ ^45^, ^46^ ^]^ with physically descriptive and computationally inexpensive analytical expressions.

Considering all the arguments introduced so far, it emerges that our findings have the potential to effectively impact practical implementations and applications. In this regard, our solutions remarkably add magnetization patterning to the designer's palette, thus extending the possibilities to program magnetic behaviors for a system. For instance, previous magnets systems for stable assemblies,^[^ ^23^ ^]^ or magnets array devised to trap magnetoresponsive agents for biomedical applications,^[^ ^13^, ^15^ ^]^ used magnets with “standard” magnetization (such as cylindrical magnets with axial magnetization, and cuboids with magnetization aligned with one edge, like popular fridge magnets), and the sought magnetic behavior was programmed through the spatial arrangement of the magnets. This approach, however, may hamper the possibility to implement compact systems, with potential negative effects, for example, on the miniaturization of tools conceived for minimally invasive biomedical procedures. Our solutions overcome this limitation, for example, by allowing to program magnetization for a given spatial arrangement. We concisely illustrated this opportunity by considering a compact cylindrical magnets array (Figure 4) aimed to trap, relatively close to the magnets, tiny agents featuring the same magnetic behavior as particles increasingly used for both diagnostic and therapeutic medical procedures.^[^ ^59^ ^]^ In particular, we showed the possibility to create, based on suitable magnetization patterns, both circumferential and helical force traps, while also modulating their global scheme and/or local features (like traps chirality for the helical ones) through magnetization re‐programming, as achievable by rotating the magnets around their common axis (Figure 4b). The considered concept array could be used, for example, to advance the design of magnetic retrieval catheters (which already demonstrated some potential for translation^[^ ^13^ ^]^), in particular toward operation in complex/swirling biological flows, yet stronger claims can only be made on the basis of physically representative flow conditions and clinically relevant implementation constraints. Furthermore, in the considered illustration we did not determine specific magnetization patterns by solving an inverse problem, that is, we did not use the achieved solutions for design in the strict sense. A subsequent design study, focused on the creation of a real magnets system with programmed magnetization such as to enable new functions/applications compared to existing magnets/coils systems, will provide more tangible evidence of the advantages of our analytical solutions for design. To the purpose, once identified specifications and constraints relevant to a real‐world problem (for instance, a biomedical procedure lacking of suitable tools), a design/optimization framework will be defined, where to integrate our solutions, starting from the self‐contained implementation that is reported, for the benefit of a wider readership, in the present paper. However, to keep the discussion scientifically focused, we skip speculations at this stage and simply underline that, even for design, our findings can provide significant advantages compared to numerical simulations. Indeed, considering cylindrical magnets systems with programmable magnetization, our exact solutions inexpensively determine the results to which numerical simulations tend, provided that the involved discretization is refined enough (Figure 2c,d; Figure 3b; Figure 4c). (In this regard, it should be noticed that our solutions can also be used as benchmarks for developing related numerical approaches.) Yet accurately resolving magnetic field variations, in particular close to the surface/edges of the magnets, requires a relatively fine discretization per se, thus further adding to computational costs, so that our solutions offer a very strong computational gain (about 10^6^), versus finite element simulations, already for the aforementioned magnets array system. Consistently, physics‐informed meshing and, possibly, gradient computation could hamper the development of design approaches based on numerical simulations, since memory requirements and computation times could rapidly become unsustainable, at least on common computing platforms, for increasingly complex systems. Conversely, our analytical solutions could enable scalable design frameworks.

To conclude, our study was focused on cylindrical magnets systems with generic uniform magnetization, for which we achieved exact and computationally robust solutions for magnetic field, gradient, force and torque. In order to tackle more articulated systems and ambitious real‐world implementations, it is necessary to co‐develop and synergistically integrate complementary contributions, for example, concerning novel magnetic materials (including soft magnetoresponsive composites and architected/foldable magnetic materials),^[^ ^57^, ^69^, ^70^, ^71^ ^]^ modeling/simulation (for both rigid magnets and magnetoelastic continua),^[^ ^72^, ^73^ ^]^ and system assembly/integration.^[^ ^74^, ^75^ ^]^ Nevertheless, original approaches and solutions grounded on classical physics knowledge continue to unfold new research pathways and potential applications,^[^ ^76^ ^]^ and, in this spirit, the analytical solutions determined in this study can be effectively used to scientifically investigate, develop, and possibly invent complex cylindrical magnets systems with programmable magnetization, for a wide variety of applications.

## 4 Methods

### Base Cylindrical Magnet and Coordinate Systems

With reference to Figure 1a, a cylindrical magnet was considered with radius $\bar{R}$, half‐height $\bar{L}$, and generic uniform magnetization **M**_⋆_. S_b_, S_t_, and S_l_ respectively denote the bottom, top, and lateral surface of the cylinder; τ_b_, τ_t_ and τ_l_ respectively denote the corresponding prolongations. Given the direction ${\hat{e}}_{∥}$ of the cylinder axis, it was defined $M_{∥}\;:=\;\left(M_{★}\cdot {\hat{e}}_{∥}\right)\;{\hat{e}}_{∥}\;:=\;M_{∥}\;{\hat{e}}_{∥}$ and $M_{⊥}\;:=\;M_{★}-M_{∥}$ such that $M_{⊥}\;:=\;||M_{⊥}\left|\right|\;{\hat{e}}_{⊥}\;:=\;M_{⊥}{\hat{e}}_{⊥}$ (without loss of generality, since for ||**M**_⊥_|| = 0 the diametric direction ${\hat{e}}_{⊥}$ can be arbitrarily chosen in the plane perpendicular to ${\hat{e}}_{∥}$).

By measuring the angular coordinate from ${\hat{e}}_{⊥}$, a cylindrical coordinate system was introduced with unit vectors $\left\{{\hat{e}}_{\rho },{\hat{e}}_{\phi },{\hat{e}}_{z}\;:=\;{\hat{e}}_{∥}\right\}$ and origin **O** at cylinder mid‐height. In this system, a point **P**′ on the cylinder surface has coordinates (${\bar{\rho }}^{\prime }$, ϕ′, ${\bar{z}}^{\prime }$), whereas a point **P** either inside or outside the cylinder has coordinates ($\bar{\rho }$, ϕ, $\bar{z}$). In order to preserve physical consistency during the derivation, the corresponding non‐dimensional coordinates ($\rho^{\prime }\;:=\;{\bar{\rho }}^{\prime }/\bar{R}$, ϕ′, $z^{\prime }\;:=\;{\bar{z}}^{\prime }/\bar{R}$) and ($\rho \;:=\;\bar{\rho }/\bar{R}$, ϕ, $z\;:=\;\bar{z}/\bar{R}$) were introduced, which are illustrated in Figure 1b together with the scaled magnet dimensions $R\;:=\;\bar{R}/\bar{R}\;=\;1$ and $L\;:=\;\bar{L}/\bar{R}$. Spatial derivatives were consistently carried out (so that, for instance, ∂_ρ_(·) ≔ ∂(·)/∂ρ and $\partial_{\bar{\rho }}\left(\cdot \right)\;:=\;\partial \left(\cdot \right)/\partial \bar{\rho }=\partial_{\rho }\left(\cdot \right)/\bar{R}$). Cylindrical coordinates were used to obtain field and gradient components, which were later recombined to represent the solution in the intrinsic (Cartesian) frame $\left\{{\hat{e}}_{x},{\hat{e}}_{y},{\hat{e}}_{z}\right\}\;:=\;\left\{{\hat{e}}_{⊥},{\hat{e}}_{∥}\times {\hat{e}}_{⊥},{\hat{e}}_{∥}\right\}$ in order to circumvent the indeterminacy of ${\hat{e}}_{\rho }$ and ${\hat{e}}_{\phi }$ (due to ϕ) for points **P** on the cylinder axis.

### Solution Procedure for Magnetic Field and Gradient

Hereafter, the solution strategy is first outlined. To obtain **H** and grad(**H**) at a generic point **P**, the magnetostatics governing equations (in the absence of free currents) curl(**H**) = **0** and div(**B**) = 0 were first recalled, where the magnetic induction **B** is locally linked to **H** and to the magnetization **M** by **B** = μ_0_(**H** + **M**), with μ_0_ ≔  4π · 10^−7^ TmA^−1^ denoting vacuum magnetic permeability.^[^ ^39^ ^]^ Once introduced the magnetic scalar potential φ such that

$$H:=-\text{grad}\left(\varphi \right),$$

a single governing equation remains, namely Δφ = div(**M**) (where Δ denotes the Laplace operator), and its solution, given the assumed uniform magnetization **M**_⋆_, formally reads^[^ ^39^ ^]^

$$\varphi \left(P\right)=\frac{1}{4\pi }\int_{S_{t}\cup S_{b}\cup S_{l}}\frac{M_{★}\cdot \hat{n}}{||P-P^{\prime }\left|\right|}\;dS^{\prime },$$

where $\hat{n}$ denotes the (outer) normal at the running point **P**′ on the cylinder surface. Then, after recasting the magnetic scalar potential as $\varphi =\left(M_{∥}\;{\tilde{\varphi }}_{∥}+M_{⊥}{\tilde{\varphi }}_{⊥}\right)\bar{R}/\left(4\pi \right)$, with

$$\begin{array}{ccc} & & {\tilde{\varphi }}_{∥}\left(P\right):={\bar{R}}^{-1}\left(\int_{S_{t}}\frac{dS^{\prime }}{||P-P^{\prime }\left|\right|}-\int_{S_{b}}\frac{dS^{\prime }}{||P-P^{\prime }\left|\right|}\right)\;\mathrm{and}\\ & & {\tilde{\varphi }}_{⊥}\left(P\right):={\bar{R}}^{-1}\int_{S_{l}}\frac{\cos\left(\phi^{\prime }\right)\;dS^{\prime }}{||P-P^{\prime }\left|\right|}, \end{array}$$

an explicit exact solution was obtained for both ${\tilde{\varphi }}_{∥}$ and ${\tilde{\varphi }}_{⊥}$, by direct integration (systematically working in non‐dimensional terms). **H** = **H**_∥_ + **H**_⊥_ and grad(**H**) = grad(**H**_∥_) + grad(**H**_⊥_) were finally achieved by computing $H_{∥}\;:=\;\left(-\text{grad}\left({\tilde{\varphi }}_{∥}\right)\right)M_{∥}\bar{R}/\left(4\pi \right)$ and $H_{⊥}\;:=\;\left(-\text{grad}\left({\tilde{\varphi }}_{⊥}\right)\right)M_{⊥}\bar{R}/\left(4\pi \right)$.

Hereafter, the solution procedure is further sketched. It consists of four main steps (Steps 1–4). Lower‐level details are fully reported in Supporting Information, for ease of readability.

At Step1, the starting expressions of ${\tilde{\varphi }}_{∥}$ and ${\tilde{\varphi }}_{⊥}$ were reworked by using Bessel functions (Section S2, Supporting Information).

At Step2, ${\tilde{\varphi }}_{∥}$ and ${\tilde{\varphi }}_{⊥}$, as well as relevant spatial derivatives thereof (up to the second order), were expressed in terms of integrals involving products of Bessel functions. Specifically, by considering that the expression of ${\tilde{\varphi }}_{⊥}$ obtained at Step1 depends on whether z ⩾ L, |z| < L or z ⩽ −L, these three cases were individually addressed (Sections S3–S5, Supporting Information).

At Step3, the derivatives obtained at Step2 were recast in terms of the Bulirsch integral C, so as to finally achieve the sought components of **H** and grad(**H**) in the cylindrical frame. To the purpose, we used C to also compute the so‐called Normalized Heuman Lambda function Λ (Section S1, Supporting Information), since its introduction allowed us to circumvent singularities that arise with some formulations involving complete elliptic integrals of the third kind.^[^ ^48^, ^49^ ^]^ More specifically, at Step3 the components of **H**_∥_ and **H**_⊥_ in Table 1 were first obtained; H_∥ϕ_ = 0 (by symmetry) was omitted for conciseness from the considered table. Then, a matrix representation for grad(**H**) was addressed, since the force **f** = μ_0_ grad(**H**) · **m** acting on a magnetic dipole **m** subject to **H** ^[^ ^1^, ^2^ ^]^ is commonly expressed through the associated matrix representation [f_ρ_, f_ϕ_, f_z_]^*T*^ = μ_0_ *G* ^(*cyl*)^ [m_ρ_, m_ϕ_, m_z_]^*T*^, with

$$G^{\left(cyl\right)}:=\left[\begin{array}{ccc} \partial_{\bar{\rho }}H_{\rho } & {\bar{\rho }}^{-1}\;\left(\partial_{\phi }H_{\rho }-H_{\phi }\right) & \partial_{\bar{z}}H_{\rho }\\ \partial_{\bar{\rho }}H_{\phi } & {\bar{\rho }}^{-1}\;\left(\partial_{\phi }H_{\phi }+H_{\rho }\right) & \partial_{\bar{z}}H_{\phi }\\ \partial_{\bar{\rho }}H_{z} & {\bar{\rho }}^{-1}\;\left(\partial_{\phi }H_{z}\right) & \partial_{\bar{z}}H_{z} \end{array}\right].$$

The derivatives of **H**_∥_ and **H**_⊥_ in Table 2 were thus obtained; ${\bar{\rho }}^{-1}\left(\partial_{\phi }H_{∥\rho }\;-\;H_{∥\phi }\right)=0$ and ${\bar{\rho }}^{-1}\partial_{\phi }H_{∥z}=0$ (by symmetry) were omitted for conciseness from the considered table. Only five *G* ^(*cyl*)^ components were shown in Table 2 because the remaining ones are obtained from the curl‐free and divergence‐free conditions, namely ${\bar{\rho }}^{-1}\partial_{\bar{\rho }}\left(\bar{\rho }\;H_{\rho }\right)+{\bar{\rho }}^{-1}\partial_{\phi }H_{\phi }+\partial_{\bar{z}}H_{z}=0$, ${\bar{\rho }}^{-1}\partial_{\phi }H_{z}=\partial_{\bar{z}}H_{\phi }$, $\partial_{\bar{z}}H_{\rho }=\partial_{\bar{\rho }}H_{z}$ and $\partial_{\bar{\rho }}\left(\bar{\rho }\;H_{\phi }\right)=\partial_{\phi }H_{\rho }$. Additional details are reported in Sections S3–S5 (Supporting Information).

At Step4, the solution was extended so as to circumvent the indeterminacy of ${\hat{e}}_{\rho }$ and ${\hat{e}}_{\phi }$ (due to ϕ) on the cylinder axis, thus achieving the complete solutions for **H** and grad(**H**). As for **H**, by representing ${\hat{e}}_{\rho }$ and ${\hat{e}}_{\phi }$ in terms of ${\hat{e}}_{x}\;=\;{\hat{e}}_{⊥}$ and ${\hat{e}}_{y}\;=\;{\hat{e}}_{∥}\;\times \;{\hat{e}}_{⊥}$ (while having ${\hat{e}}_{z}\;=\;{\hat{e}}_{∥}$), $H\;=\;\left(H_{∥\rho }+H_{⊥\rho }\right)\;{\hat{e}}_{\rho }+\left(H_{∥\phi }+H_{⊥\phi }\right)\;{\hat{e}}_{\phi }+\left(H_{∥z}+H_{⊥z}\right)\;{\hat{e}}_{z}$ was recast by only using ${\hat{e}}_{∥}$ and ${\hat{e}}_{⊥}$, and that expression was manipulated so as to finally achieve the compact frameless expression in Equation 6. As for grad(**H**), the matrix representation of the above force **f** in the intrinsic frame was considered, namely [f_x_, f_y_, f_z_]^*T*^ = μ_0_ *G* [m_x_, m_y_, m_z_]^*T*^, with

$$G:=\left[\begin{array}{ccc} \partial_{\bar{x}}H_{x} & \partial_{\bar{y}}H_{x} & \partial_{\bar{z}}H_{x}\\ \partial_{\bar{x}}H_{y} & \partial_{\bar{y}}H_{y} & \partial_{\bar{z}}H_{y}\\ \partial_{\bar{x}}H_{z} & \partial_{\bar{y}}H_{z} & \partial_{\bar{z}}H_{z} \end{array}\right],$$

and the compact solution in Equation 15 was obtained by computing $G\;=\;R\;G^{\left(cyl\right)}\;R^{-1}$ via the rotation matrix R:=[cosϕ,−sinϕ,0;sinϕ,cosϕ,0;0,0,1] (semicolon here denoting row splitting) that maps the cylindrical representation into that one in the intrinsic frame. Additional details are reported in Section S6 (Supporting Information).

Exact analytical solutions for **H** and grad(**H**) were thus finally achieved, which do not suffer from singularities in the whole computational domain. Selected components were also illustrated (Figure 2b–d) by computing the solution on a surface S obtained by cutting in half the cylindrical surface $5\cdot 10^{-2}\bar{R}$ inward from the magnet surface. The exact solution for the underlying magnetic scalar potential φ was also achieved, which is reported in Section S7 (Supporting Information), for completeness.

### Solution Procedure for Magnetic Force and Torque

Two coaxial cylindrical magnets C1 (radius ${\bar{R}}_{1}$, half‐height ${\bar{L}}_{1}$, magnetization **M**_1_) and C2 (radius ${\bar{R}}_{2}$, half‐height ${\bar{L}}_{2}$, magnetization **M**_2_) were considered, at a relative distance $\bar{d}\;\geq \;{\bar{L}}_{1}\;+\;{\bar{L}}_{2}$. Force and torque exerted by C1 on C2 can be respectively computed as follows:^[^ ^38^ ^]^

$$\begin{array}{c} \begin{array}{cc} f^{1\to 2}=\; & \mu_{0}\int_{V_{2}}\text{grad}\left(H_{1}\right)\;\cdot \;M_{2}\;dV,\\ t^{1\to 2}=\; & \mu_{0}\int_{V_{2}}\;\left(\left(P\;-\;O_{1}\right)\times \left(\text{grad}\left(H_{1}\right)\cdot M_{2}\right)+M_{2}\times H_{1}\right)\;dV, \end{array} \end{array}$$

where (subscripts are understood and) *V*_2_ denotes the volume occupied by C2. First, by assuming axial magnetizations **M**_1_ = **M**_∥1_ and **M**_2_ = **M**_∥2_, the associated force $f_{∥}^{1\to 2}$ was computed (torque being null, by symmetry). Then, by assuming diametric magnetizations **M**_1_ = **M**_⊥1_ and **M**_2_ = **M**_⊥2_ at a generic relative angular shift θ, the corresponding force $f_{⊥}^{1\to 2}$ and torque $t_{⊥}^{1\to 2}$ were computed. More specifically, given the axial direction ${\hat{e}}^{1\to 2}$ pointing from C1 to C2, force and torque were represented in the intrinsic frame associated with C1, by assuming ${\hat{e}}_{z}\;=\;{\hat{e}}_{∥}\;=\;{\hat{e}}^{1\to 2}$ (without loss of generality). Once consistently adopted ${\bar{R}}_{1}$ as reference length for non‐dimensionalization, **H**_1_ and grad(**H**_1_) were replaced with the exact expressions previously achieved for field and gradient, respectively, and integration about the axial direction was directly performed, ending up with the same kind of integrals obtained at Step2 of the solution procedure for field and gradient. Proceeding as at Step3, the expressions at hand were then recast in terms of C, thus finally reaching the results in Table 3, which seamlessly account for the three cases ${\bar{R}}_{2}⋚{\bar{R}}_{1}$ through a unified expression, and do not suffer from singularities even in the limit case of magnets in contact with each other. Additional details are reported in Section S8 (Supporting Information).

### Magnets Array Actuation System

It was assumed to have *n*_*r*_ = 6 ring arrays, each containing *n*_*m*_ = 6 evenly spaced magnets (Figure 4a), with $\bar{R}\;=\;2\;\cdot \;10^{-3}\;m$, L = 1 and ||**M**_⋆_|| = 10^6^Am^−1^ (as for common NdFeB magnets), around a cylindrical workspace I having radius $2.5\bar{R}$ and axial span $L_{I}\bar{R}\;=\;13.5\bar{R}$. In order to investigate a compact array embodiment, the rings radius (namely the distance from the workspace axis to the center **O** of each magnet) was set equal to $3.75\bar{R}$, and the magnets were evenly distributed along the axial span (thus resulting in a $\left(L_{I}\;-\;2\;L\right)\bar{R}/\left(n_{r}\;-\;1\right)$ axial shift between adjacent rings). Furthermore, all the magnets were aligned with the workspace axial direction, by setting ${\hat{e}}_{∥}\;=\;{\hat{e}}_{w}$, and an angular shift of π/*n*_*m*_ rad between adjacent rings was introduced. Finally, for each magnet, the magnetization orientation was parameterized via the polar angle λ ∈ [0, π] rad and the azimuthal angle ς ∈ [ − π, π] rad shown in Figure 4a (with ς defining an angular offset with respect to the sagittal plane passing through the workspace axis and **O**).

At each point on the workspace lateral boundary, the axial force $f_{w}\;:=\;f\;\cdot \;{\hat{e}}_{w}$ was computed by assuming the presence of a spherical superparamagnetic agent, modeled as an induced dipole **m** = κ**H** (by neglecting magnetization saturation effects possibly limiting dipole moment intensity^[^ ^2^, ^12^, ^28^ ^]^), and the normalized axial force was then obtained as ${\tilde{f}}_{w}\;:=\;f_{w}\bar{R}/(\mu_{0}\;||M_{★}\left||\;||m||\right)$ (so that κ, above introduced to define **m** in a physically consistent way, is immaterial), with the trivial extension ${\tilde{f}}_{w}\;:=\;0$ for ||**m**|| = 0. In order to compute ${\tilde{f}}_{w}$, we implemented the exact solutions achieved for **H** and grad(**H**) in Matlab (The MathWorks, USA), by accounting for the array structure via computationally inexpensive superposition. Finally, starting from the nodal force lines ${\tilde{f}}_{w}\;=\;0$ on the workspace lateral surface, magnetic traps were defined in correspondence of negative values of the directional derivative of ${\tilde{f}}_{w}$ along ${\hat{e}}_{w}$. Let us remark that the as‐computed traps are not affected by magnetization saturation effects, consistently with the expression introduced above for the induced point dipole.

### Comparison with Numerical Simulations and Dipole Approximation

The obtained exact analytical results were compared to numerical simulations carried out by means of the finite‐element solver Comsol Multiphysics (Comsol Inc, USA). Simulations were run on a desktop personal computer (CPU: 3 GHz; RAM: 128 GB), by assuming $\bar{R}\;=\;10^{-2}\;m$ and ||**M**_⋆_|| = 10^6^Am^−1^.

As for **H** and grad(**H**) of a single magnet, two test‐cases were considered, namely L = 1/2 and L = 2. For each of them, 46 simulations were run, by parameterizing the magnetization orientation through 46 evenly spaced values of λ ∈ [0, π/2] rad, with cos λ = M_∥_/||**M**_⋆_|| (Figure 2a; half of the [0, π] span was considered, for simplicity). The magnetostatics governing equations were solved in a spherical domain centered at **O**, by imposing φ = 0 on its boundary (far enough from **O** not to affect the solution, as ex post verified). The domain was discretized by 2nd‐order‐accurate tetrahedral elements, and the grid was incrementally refined to obtain discretization‐independent results. For each simulation, the (vector) numerical solution N was exported at grid points within a cube centered at **O** and having (non‐dimensional) edge length 6, by neglecting grid points within a 5 · 10^−2^‐thin shell around the magnet surface. Once computed the analytical solution A at the same grid points by using Matlab (The MathWorks, USA), the relative difference ${\Sigma (|\left(\cdot \right)}_{A}-{\left(\cdot \right)}_{N}|)\;/{\;\Sigma (|\left(\cdot \right)}_{N}\left|\right)$ was computed, where (·) represents a generic field or gradient component, Σ denotes numerical integration over the cube (to account for the uneven spatial distribution of grid points), and subscripts are understood. Analytical and simulation results were also visually compared (Figure 2c,d), by reporting selected components on the following cut‐lines (relatively close to the magnet): CL1 ≔ {|x| ⩽ 3/2, y = 0, z = L + 1/10}, CL2 ≔ {x = 11/10, y = 0, |z| ⩽ L + 1/2}, $\mathrm{CL}3\;:=\;\{x\;=\;11\sqrt{2}/20,y\;=\;11\sqrt{2}/20,|z|\;\leq \;L\;+\;1/2\}$, and CL4 ≔ {x = 0, y = 11/10, |z| ⩽ L + 1/2}.

Moreover, as regard force and torque, it was preliminarily verified that the expression for $f_{∥}^{1\to 2}$ produces the same results as the expression in ref. [55], so as to focus on $f_{⊥}^{1\to 2}$ and $t_{⊥}^{1\to 2}$, for which analytical benchmarks are not available. Once fixed L_1_ = L_2_ = 1 and the same magnetization intensity ||**M**_⋆_|| for both magnets, two test‐cases were considered, namely R_2_ = 1/2 and R_2_ = 2. For each of them, we ran 2 × 30 = 60 simulations. Specifically, 2 values were chosen for the face‐to‐face distance $\tilde{d}\;:=\;d-L_{1}-L_{2}$, namely 1/10 and 3/2, and for each of them the angular shift between the involved magnetizations was parameterized through 30 evenly spaced values of θ ∈ [0, π] rad (Figure 3a; half of the [0, 2π] span was considered, for simplicity). Discretization was performed in a similar way as described above. For each simulation, the resulting (scalar) numerical values for force and torque, say ${\left(\cdot \right)}_{N}$ to leverage the above notation, were exported, and the corresponding analytical values ${\left(\cdot \right)}_{A}$ were computed. For each value of $\tilde{d}$, the relative difference $\mathrm{RMS}\left({\left(\cdot \right)}_{A}-{\left(\cdot \right)}_{N}\right)\;/\;\mathrm{RMS}\left({\left(\cdot \right)}_{N}\right)$ was finally computed, with the root mean square (RMS) value defined over each (evenly distributed) θ−set. Analytical and simulation results were also visually compared (Figure 3b), by reporting the normalized force $f_{⊥}\;:=\;\left(f_{⊥}^{1\to 2}\cdot {\hat{e}}^{1\to 2}\right)/(\mu_{0}\;{\bar{R}}_{1}\;{\bar{R}}_{2}\;||M_{★}{\left|\right|}^{2})$ and torque $t_{⊥}\;:=\;\left(t_{⊥}^{1\to 2}\cdot {\hat{e}}^{1\to 2}\right)/(\mu_{0}\;{\bar{R}}_{1}^{2}\;{\bar{R}}_{2}\;||M_{★}{\left|\right|}^{2})$.

Furthermore, as concerns the magnets array actuation system, the (λ = π/4, ς = π/2) magnetization pattern was considered. A spherical domain was defined, centered around the array workspace and large enough for the null‐potential boundary condition not to affect the solution, as mentioned above. The domain was discretized by the aforementioned tetrahedral elements, and the grid was incrementally refined to obtain discretization‐independent results, by paying careful attention to the boundary layers close to the surface of each magnet (and in particular to the edges), where the solution undergoes relatively sharp variations. The number of elements used for the considered test‐case (around 80 · 10^6^), as well as those used for the aforementioned simpler simulations (nearly 10 · 10^6^), are consistent, for example, with the number of elements (around 2 · 10^6^) needed to simulate a simple cylindrical tile.^[^ ^61^ ^]^ For each field component, we exported the numerical solution at evenly spaced points along four cutlines on the lateral surface of the workspace (denoted as CL5‐CL8 in Figure 4c), so as to compute the associated RMS relative difference with respect to the analytical solution, as described above. More in detail, the axial cutlines CL5 and CL6 were introduced by intersecting the workspace lateral surface with sagittal planes passing through the workspace axis and containing the origins of axially aligned magnets. Considering symmetry, CL5 and CL6 were chosen as defined by $\phi_{I}\;=\;0$ and $\phi_{I}\;=\;\pi /n_{m}\;\text{rad}$, respectively, with the reference direction for the angular coordinate $\phi_{I}$ defined (up to *n*_*m*_ angular shifts, by symmetry) in Figure 4c. The circumferential cutlines CL7 and CL8 were defined by intersecting the workspace lateral surface with planes perpendicular to the workspace axis. Considering symmetry, CL7 was defined by picking the plane bisecting the workspace axial span, and CL8 by picking the plane through the origins of the magnets in a ring adjacent to the aforementioned bisecting plane. Analytical and simulation results were also visually compared (Figure 4c).

Finally, the obtained exact solutions were also compared with the results provided by the dipole approximation. As for **H** and grad(**H**) of a single magnet, the aforementioned selected components were computed on CL1‐CL4 by using the well‐known dipole expressions.^[^ ^39^ ^]^ Considering that the multipole expansion is well defined for points **P** outside the smallest sphere bounding the magnet,^[^ ^39^ ^]^ the cut‐line segments that intersect that sphere were identified, so as to highlight those intervals (gray in Figure 2c,d) where the dipole approximation was expected to deteriorate (differently from the exact solution, which does not suffer from any limitations). Using the well‐known expressions for force and torque between dipoles,^[^ ^39^ ^]^ the relative error on $\left|\right|f_{⊥}^{1\to 2}\left|\right|$ and $\left|\right|t_{⊥}^{1\to 2}\left|\right|$ (with respect to the exact solutions) was then computed, by varying $\tilde{d}$ and R_2_ (resp. L_2_), with L_2_ = 1 (resp. R_2_ = 2). To the purpose, it was also fixed θ so as to maximize force and torque intensity, for ease of rendering. Consistently with above, those regions in the R_2_‐$\tilde{d}$ (resp. L_2_‐$\tilde{d}$) plane were highlighted (transparent white layer in Figure 3c) where the dipole approximation was expected to deteriorate (differently from the exact solution, which does not suffer from any limitations), because at least one portion of a magnet intersects the smallest sphere bounding the other one.

### Solution Implementation

The exact solutions for magnetic field and gradient reported in Tables 1 and 2, respectively, can be fully implemented by using the self‐contained set of expressions in **Table** **4**. Specifically, group FG1 reports the coordinates‐related definitions (while also including ρ and z for ease of readability). In the computational domain, which excludes the magnet surface (whence the edges), $0\;\leq \;\sigma_{\pm }^{2}\;\leq 1$ and $0\;\leq \;k_{\pm }^{2}\;<\;1$. Group FG2 then reports the so‐called Normalized Heuman Lambda function Λ, consistently defined (in terms of C) for 0 ⩽ σ^2^ ⩽ 1 and 0 ⩽ *k* < 1 (Λ can be robustly computed even for σ^2^ = 1, see Section S1, Supporting Information). Moreover, group FG3 defines the functions *f*_0_ − *f*_5_ appearing in Tables 1 and 2, by also using sign and H to denote sign and Heaviside step functions, respectively. Function *f*_2_, in particular, can be robustly computed even on the magnet axis, namely for ρ = 0 (see Sections S3–S5, Supporting Information). Furthermore, function $f_{\Lambda }$, which seamlessly accounts for the three cases z ⩾ L, |z| < L and z ⩽ −L, (see Sections S2–S5, Supporting Information), is continuous across the surfaces τ_b_ and τ_t_ shown in Figure 1a (whereas function *f*_0_ simply accounts for magnetization discontinuity when crossing the corresponding surfaces S_b_ and S_t_). Finally, group FG4 and FG5 report key definitions for the final, complete solutions: FG4 defines **u** and **v** appearing in Equation 6, whereas FG5 defines ${\tilde{J}}_{∥}$ and ${\tilde{J}}_{⊥}$ featuring in Equation 15.

**Table 4:** Implementation of the exact solutions for magnetic field and gradient

Group	Auxiliary expressions
FG1	$p\;:=\;\left(P\;-\;O\right)\;/\bar{R},\;\rho =∥p\;\times \;{\hat{e}}_{∥}∥,\;z=p\;\cdot \;{\hat{e}}_{∥},\;s_{\phi }:=\sin\phi ,\;c_{\phi }:=\cos\phi ,\;z_{\pm }:=z\pm L,\;\sigma_{\pm }^{2}:=z_{\pm }^{2}/\left({\left(1\;-\;\rho \right)}^{2}+z_{\pm }^{2}\right)$ $d_{\pm }^{2}:={\left(1\;+\;\rho \right)}^{2}+z_{\pm }^{2},\;d_{\pm }:=\sqrt{d_{\pm }^{2}},\;k_{\pm }^{2}:=4\rho /d_{\pm }^{2},\;k_{\pm }:=\sqrt{k_{\pm }^{2}},\;k_{c\pm }^{2}:=1-k_{\pm }^{2},\;k_{c\pm }:=\sqrt{k_{c\pm }^{2}}$
FG2	$\Lambda \left(\sigma^{2},k\right):=\sqrt{\tilde{p}\;\sigma^{2}}\;C\left(k_{c},\tilde{p},1,k_{c}^{2}\right),\;\mathrm{with}\;\tilde{p}:=\left(1\;-\;\sigma^{2}k_{c}^{2}\right)/\left(1\;-\;\sigma^{2}\right),\;k_{c}^{2}\;:=\;1\;-\;k^{2},\;k_{c}\;:=\;\sqrt{k_{c}^{2}}$
FG3	$f_{1}\left(\rho ,z;L\right):=\frac{1}{4}\left({\left[\frac{z_{i}}{d_{i}}\;C\left(k_{ci},1,1,1\right)\right]}_{-}^{+}\;+f_{\Lambda }\right),\;f_{2}\left(\rho ,z;L\right):=\frac{\rho^{-3}}{4}\left({\left[\frac{z_{i}}{d_{i}}\;C\left(k_{ci},1,1\;-\;2\rho ,1\;+\;2\rho \right)\right]}_{-}^{+}\;-f_{\Lambda }\right)$ $f_{3}\left(\rho ,z;L\right):=4{\left[\frac{1}{d_{i}^{3}}\;C\left(\frac{2\sqrt{k_{ci}}}{1+k_{ci}},1,0,\frac{2}{{\left(1+k_{ci}\right)}^{3}}\right)\right]}_{-}^{+},\;f_{4}\left(\rho ,z;L\right):={\left[\frac{z_{i}}{d_{i}^{3}}\;C\left(k_{ci},1,\frac{1}{k_{ci}^{2}},-1\right)\right]}_{-}^{+}$ $f_{5}\left(\rho ,z;L\right):={\left[\frac{1}{d_{i}^{3}}\;C\left(k_{ci},1,\frac{1\;-\;\rho }{k_{ci}^{2}},1\;+\;\rho \right)\right]}_{-}^{+},\;{\left[\;f(z_{i},d_{i},k_{i},\text{…})\right]}_{-}^{+}:=f\left(z_{+},d_{+},k_{+},\text{…}\right)-f\left(z_{-},d_{-},k_{-},\text{…}\right)$ $$\left\{\begin{array}{ccc} f_{\Lambda }\left(\rho ,z;L\right):=\;\;\mathrm{sign}\left(1\;-\;\rho \right){\left[\Lambda (\sigma_{i}^{2},k_{i})\right]}_{-}^{+}, & f_{0}\left(\rho ,z;L\right):=0 & \;\;\mathrm{for}\;\;z\geq L\\ f_{\Lambda }\left(\rho ,z;L\right):=\;\;\mathrm{sign}\left(1\;-\;\rho \right)\left(\Lambda \left(\sigma_{+}^{2},k_{+}\right)+\Lambda \left(\sigma_{-}^{2},k_{-}\right)\right), & f_{0}\left(\rho ,z;L\right):=-\pi \;H\;\left(1\;-\;\rho \right) & \;\;\mathrm{for}\;\;|z|<L\\ f_{\Lambda }\left(\rho ,z;L\right):=\;\;\mathrm{sign}\left(1\;-\;\rho \right){\left[\Lambda (\sigma_{i}^{2},k_{i})\right]}_{+}^{-}, & f_{0}\left(\rho ,z;L\right):=0 & \;\;\mathrm{for}\;\;z\leq -L \end{array}\right.$$
FG4	$$u:=\rho \;\left(M_{⊥}\;-2\;\left(M_{⊥}\;\cdot \;\nu \right)\;\nu \right),\;v\;:=\;\left(p\cdot (M_{∥}\;-\;M_{⊥})\right)\;{\hat{e}}_{∥}-M_{∥}\;p,\;\left\{\begin{array}{cc} \nu :=(p\;\times \;{\hat{e}}_{∥})/\rho & \;\mathrm{for}\;\;\rho >0\\ \nu :=0 & \;\mathrm{for}\;\;\rho =0 \end{array}\right.$$
FG5	$$\left\{\begin{array}{ccc} {\tilde{J}}_{∥}:=J_{∥},\;{\tilde{J}}_{⊥}:=J_{⊥},\;\tilde{c}:=\left(p\cdot {\hat{e}}_{⊥}\right)/\rho , & \tilde{s}:=\left(p\cdot {\hat{e}}_{∥}\times {\hat{e}}_{⊥}\right)/\rho & \mathrm{for}\;\;\rho >0\\ {\tilde{J}}_{∥}:={\tilde{J}}_{∥}^{0},\;{\tilde{J}}_{⊥}:={\tilde{J}}_{⊥}^{0},\;\tilde{c}:=0, & \tilde{s}:=0 & \mathrm{for}\;\;\rho =0 \end{array}\right.$$ $$J_{∥}:=\left[\begin{array}{ccc} g_{1}-f_{3} & \;g_{2} & \;g_{4}\\ g_{2} & \;g_{3}-f_{3} & \;g_{5}\\ g_{4} & \;g_{5} & \;f_{5} \end{array}\right]\;,\;\;\;J_{⊥}:=\left[\begin{array}{ccc} \tilde{c}\;\left(6f_{2}-{\tilde{c}}^{2}g_{6}\right) & \;\tilde{s}\;\left(2f_{2}-{\tilde{c}}^{2}g_{6}\right) & \;g_{1}-f_{3}\\ \tilde{s}\;\left(2f_{2}-{\tilde{c}}^{2}g_{6}\right) & \;\tilde{c}\;\left(2f_{2}-{\tilde{s}}^{2}g_{6}\right) & \;g_{2}\\ g_{1}-f_{3} & \;g_{2} & \;g_{4} \end{array}\right]\;,\;\;\;J_{∥}^{0}:=\left[\begin{array}{ccc} -g_{★} & \;0 & \;0\\ 0 & \;-g_{★} & \;0\\ 0 & \;0 & \;2g_{★} \end{array}\right]\;,\;\;\;J_{⊥}^{0}:=\left[\begin{array}{ccc} 0 & \;0 & \;-g_{★}\\ 0 & \;0 & \;0\\ -g_{★} & \;0 & \;0 \end{array}\right]$$ $g_{1}:={\tilde{c}}^{2}\;g\;,\;g_{2}:=\tilde{s}\;\tilde{c}\;g\;,\;g_{3}:={\tilde{s}}^{2}\;g\;,\;g_{4}:=\tilde{c}\;f_{4},\;g_{5}:=\tilde{s}\;f_{4},\;g_{6}:=8f_{2}+f_{4},\;g:=2\;f_{3}-f_{5},\;g_{★}:=\frac{\pi }{4}{\left[{\left(1+z_{i}^{2}\right)}^{-\frac{3}{2}}\right]}_{-}^{+}$

The exact solutions for magnetic force and torque reported in Table 3 can be fully implemented by using the self‐contained set of expressions in **Table** **5**. Specifically, group FT1 defines the functions η_*f*_ and ζ_*t*_ appearing in Table 3. Group FT2 then reports the underlying definitions, in particular in terms of C, with *x* ⩾ 0 (for consistency with the assumption d − L_1_ − L_2_ ⩾ 0) and 0 < ξ ⩽ 1 (to seamlessly deal with the three cases ${\bar{R}}_{2}⋚{\bar{R}}_{1}$ through a single analytical expression).

**Table 5:** Implementation of the exact solutions for magnetic force and torque

Group	Auxiliary expressions
FT1	$\eta_{f}\left(R_{2},L_{1},L_{2},d\right):=\eta \left(R_{2},d+L_{1}+L_{2}\right)+\eta \left(R_{2},d-L_{1}-L_{2}\right)-\eta \left(R_{2},d-L_{1}+L_{2}\right)-\eta \left(R_{2},d+L_{1}-L_{2}\right)$ $\zeta_{t}\left(R_{2},L_{1},L_{2},d\right)\;:=\zeta \left(R_{2},d+L_{1}+L_{2}\right)+\zeta \left(R_{2},d-L_{1}-L_{2}\right)-\zeta \left(R_{2},d-L_{1}+L_{2}\right)-\zeta \left(R_{2},d+L_{1}-L_{2}\right)$
FT2	$$\left\{\begin{array}{ccc} \eta \left(R_{2},x\right):=x\left(\;f_{6}-f_{7}\right)/\;\ell , & \zeta \left(R_{2},x\right):=\left(\left(2\;\left(1+R_{2}^{2}\right)-x^{2}\right)\;f_{6}+f_{8}\right)/\;\ell & \mathrm{for}\;\;\left(R_{2},x\right)\neq \left(1,0\right)\\ \eta (R_{2},x):=0, & \zeta (R_{2},x):=4 & \mathrm{for}\;\;\left(R_{2},x\right)=\left(1,0\right) \end{array}\right.$$ $f_{6}\left(R_{2},x\right):=k\;C\left(k_{c},1,0,-1\right),\;f_{7}\left(R_{2},x\right):=C\left(k_{c},1-\xi k,\xi ,\xi -k\right)$ $f_{8}\left(R_{2},x\right):=C\left(k_{c},1-\xi k,4R_{2}+3\xi x^{2},4R_{2}+3\xi x^{2}-k\;\left(4\xi R_{2}+3x^{2}\right)\right),\;\xi :=\min\left(1,R_{2}\right)\;/\max\left(1,R_{2}\right)$ $k\left(R_{2},x\right):=R_{2}\;/\;\ell^{2},\;k_{c}\left(R_{2},x\right):=+\sqrt{1-k^{2}},\;\ell \left(R_{2},x\right):=\left(\sqrt{{\left(1+R_{2}\right)}^{2}+x^{2}}+\sqrt{{\left(1-R_{2}\right)}^{2}+x^{2}}\right)/\;2$

## Conflict of Interest

The authors declare no conflict of interest.

## Author Contributions

F.M.: Methodology (supporting); validation; software; visualization. E.S.: Conceptualization; methodology (leading); validation; software; visualization; writing; supervision.

## Supporting information

Supporting Information

## Data Availability

All relevant data are included in the Article and its Supporting Information.
