# Supplementary material for: Exact and Computationally Robust Solutions for Cylindrical Magnets Systems with Programmable Magnetization
Source: Adv Sci (Weinh). 2023 Jul 17;10(25):2301033. doi: 10.1002/advs.202301033 (PMC10477869; doi:10.1002/advs.202301033)
Supplement: Supplementary file 1 — Supporting Information [file ADVS-10-2301033-s001.pdf]

ADVANCED SCIENCE
Open Access

Supporting Information

for *Adv. Sci.*, DOI 10.1002/advs.202301033

Exact and Computationally Robust Solutions for Cylindrical Magnets Systems with Programmable Magnetization

*Federico Masiero and Edoardo Sinibaldi**

Supporting Information

for:

Exact and computationally robust solutions for cylindrical magnets systems with programmable magnetization

*Federico Masiero, Edoardo Sinibaldi**

F. Masiero
Biorobotics Institute, Scuola Superiore Sant'Anna, Pisa, Italy
Department of Excellence in Robotics and AI, Scuola Superiore Sant'Anna, Pisa, Italy

E. Sinibaldi
Istituto Italiano di Tecnologia, Genoa, Italy
Email: edoardo.sinibaldi@iit.it

Content:

- S1. The function that permits to compute all the solutions: $\mathcal{C}$
- S2. Magnetic field and gradient: Solution details for Step1
- S3. Magnetic field and gradient: Solution details for Step2-3, case $z \geq L$
- S4. Magnetic field and gradient: Solution details for Step2-3, case $|z| < L$
- S5. Magnetic field and gradient: Solution details for Step2-3, case $z \leq -L$
- S6. Magnetic field and gradient: Solution details for Step4
- S7. Magnetic scalar potential: Solution
- S8. Magnetic force and torque: Solution details
- S9. References

Note: This Supporting Information fully details the procedure leading to the exact solutions reported in the main text. For completeness, deep-level passages are also reported, for each step of the solution procedure. Moreover, in order to foster reproducibility, contextual details are added when citing some references, as appropriate for pointing to specific expressions therein. For instance, ([SR1] 19.2.11) points to expression 19.2.11 in reference [SR1]. Furthermore, equations are sequentially numbered starting from the last number appearing in the main text, for ease of readability.

S1. The function that permits to compute all the solutions: $\mathcal{C}$

All the solutions achieved in the study are computed based on the so-called Bulirsch integral $\mathcal{C}$, which is defined as follows:

$$\mathcal{C}(k_c, p, a, b) := \int_0^{\pi/2} \frac{a\,\cos^2\psi + b\,\sin^2\psi}{(\cos^2\psi + p\,\sin^2\psi)\,\sqrt{\cos^2\psi + k_c^2\,\sin^2\psi}}\,\mathrm{d}\psi, \tag{19}$$

with $k_c, p, a, b \in \mathbb{R}$, $k_c \neq 0$ and $p \neq 0$ ([SR1] 19.2.11). $\mathcal{C}$, which is commonly implemented in software libraries [SR2], is the only function that is needed to implement all the analytical solutions obtained for magnetic field, gradient, force and torque.

$\mathcal{C}$ permits to conveniently compute the complete elliptic integrals of the first, second and third kind, respectively denoted in literature by K, E and Π [SR3], as follows: $K(k) = \mathcal{C}(k_c, 1, 1, 1) = \mathcal{C}(k_c, w, 1, w)$, $E(k) = \mathcal{C}(k_c, 1, 1, k_c^2)$ and $\Pi(1-p, k) = \mathcal{C}(k_c, p, 1, 1)$, with $k_c^2 := 1 - k^2$ and $w \in \mathbb{R}$. The following relations, which were used in the derivations, are thus immediately verified: $u\,K(k) + v\,E(k) = \mathcal{C}(k_c, 1, u{+}v, u{+}v{-}vk^2)$, $u\,K(k) + v\,\Pi(1{-}p, k) = \mathcal{C}(k_c, p, u{+}v, up{+}v)$, with $u, v \in \mathbb{R}$. In order to avoid some representation singularities at $\rho = 0$ (e.g., for $\mathrm{H}_{\|\rho}/\bar{\rho}$ when computing $\mathrm{grad}\,(\mathbf{H})$ in cylindrical coordinates), we also exploited the following relation:

$$\rho^{-1}\,\mathcal{C}(k_{ci}, 1, -1, 1) = \frac{4}{\mathrm{d}_i^2}\,\mathcal{C}\left(\frac{2\sqrt{k_{ci}}}{1 + k_{ci}}, 1, 0, \frac{2}{(1 + k_{ci})^3}\right)$$

(which can be derived by applying the Gauss transformation to the underlying elliptic integrals [SR3]), in particular through the introduction of f_3 in Table 4 (from which even k_{ci} and d_i are recalled). For the sake of illustration, Figure S1a shows some contour plots of $\mathcal{C}(k_c, p, a, b)$, for selected values of p, a and b.

In order to determine the solution for magnetic field and gradient, we also used the so-called normalized Heuman Lambda function Λ, since its introduction allowed us to circumvent singularities that arise, in particular, when using Π. Λ can be defined as follows ([SR3] 150.02, up to a sign amendment consistent with 410.04): $\Lambda(\sigma^2, k) := \sqrt{\tilde{p}\,\sigma^2}\;\mathfrak{L}$, with $\tilde{p} := (1 - \sigma^2 k_c^2)/(1 - \sigma^2)$, $k_c^2 := 1 - k^2$, $\mathfrak{L} := \int_0^{K(k)} \mathrm{d}u\,\mathrm{dn}^2(u)/\,(1{-}\omega\,\mathrm{sn}^2(u))$ and $\omega{:=}1{-}\tilde{p}$. (For reference, let us observe that σ^2 corresponds to $\sin^2(\beta)$ in [SR3]). Λ is well-behaved over its domain ($0 \le \sigma^2 \le 1$, $0 \le k \le 1$), including its boundaries: $\Lambda(0, k) = 0$, $\Lambda(1, k) = \pi/2$, $\Lambda(\sigma^2, 0) = \pi\sqrt{\sigma^2}\,/2$, and $\Lambda(\sigma^2, 1) = \arcsin(\sqrt{\sigma^2})$ (see Figure S1b). However, by observing that ([SR3] 339.01) $\mathfrak{L} = \omega^{-1}\,(k^2\,K(k) + (\omega - k^2)\,\Pi(\omega, k)) = \omega^{-1}(k^2\,\mathcal{C}(k_c, \tilde{p}, 1, \tilde{p}) + (\omega{-}k^2)\,\mathcal{C}(k_c, \tilde{p}, 1, 1)) = \mathcal{C}(k_c, \tilde{p}, 1, k_c^2)$, we defined Λ via $\mathcal{C}$ as in Table 4, thus making it possible to compute all the solutions by means of a single algorithmic building block, namely $\mathcal{C}$. While enabling compact and robust implementations, this can also foster computational efficiency, e.g., by algorithmic optimization, in particular when addressing complex magnets systems.

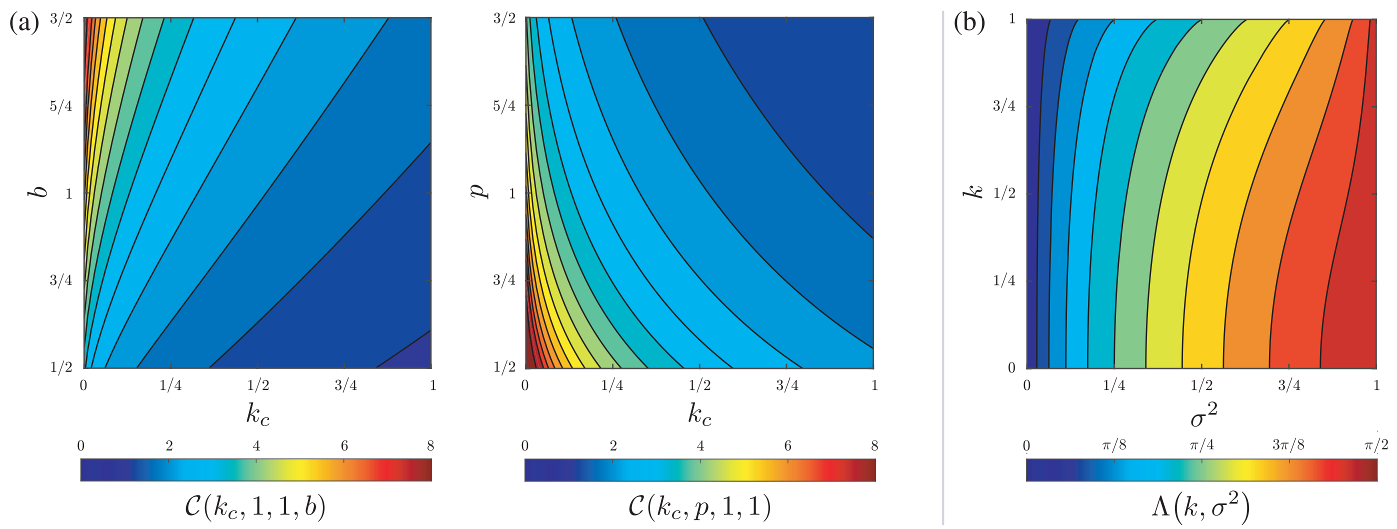

Figure S1. a) Illustrative contour plots of $\mathcal{C}(k_c, p, a, b)$, for selected values of p, a and b. b) Contour plot of $\Lambda(\sigma^2, k)$.

S2. Magnetic field and gradient: Solution details for Step1

Working in non-dimensional cylindrical coordinates (for which we systematically assumed $\rho > 0$, without loss of generality), at Step1 we expressed $\tilde{\varphi}_{\parallel}$ and $\tilde{\varphi}_{\perp}$ in terms of the Bessel functions of the first kind J_n [SR4] (by also recalling relevant expressions such as $\mathrm{z}_{\pm}$, c_ϕ and $[\cdot]_-^+$ from Table 4).

As for $\tilde{\varphi}_{\parallel}$, once introduced $\psi := \phi - \phi'$ and $\delta := +\sqrt{\rho^2 + \rho'^2 - 2\rho\rho'\, c_\psi}$, from ([SR4] 6.611-1) it follows that:

$$\frac{\bar{\mathrm{R}}}{||\mathbf{P} - \mathbf{P}'||} = \frac{1}{\sqrt{\delta^2 + \mathrm{z}_i^2}} = \int_0^\infty J_0(s\delta)\, e^{-s|\mathrm{z}_i|}\, \mathrm{d}s, \quad i = + \text{ for } \mathbf{P}' \in \mathrm{S_b}, \quad i = - \text{ for } \mathbf{P}' \in \mathrm{S_t} \tag{20}$$

(with $\mathrm{S_b}$ and $\mathrm{S_t}$ shown in Figure 1a). Moreover, considering that ([SR4] 8.531-1)

$$J_0(s\delta) = J_0(s\rho')J_0(s\rho) + 2\sum_{m=1}^{\infty} J_m(s\rho')J_m(s\rho)\, c_{m\psi}, \tag{21}$$

so that Equation (20) and (21) lead to

$$\int_0^{2\pi} \left[\frac{\mathrm{d}\phi'}{\sqrt{\delta^2 + \mathrm{z}_i^2}}\right]_+^- = 2\pi \left[\int_0^\infty J_0(s\rho')J_0(s\rho)\, e^{-s|\mathrm{z}_i|}\, \mathrm{d}s\right]_+^-,$$

one obtains

$$\frac{\tilde{\varphi}_{\parallel}}{2\pi} = \int_0^1 \mathrm{d}\rho'\rho' \left[\int_0^\infty J_0(s\rho')J_0(s\rho)\, e^{-s|\mathrm{z}_i|}\, \mathrm{d}s\right]_+^-,$$

and by using the fact that $\int_0^1 \mathrm{d}\rho'\, \rho' J_0(s\rho') = s^{-1}J_1(s)$, the sought expression for $\tilde{\varphi}_{\parallel}$ finally reads:

$$\frac{\tilde{\varphi}_{\parallel}}{2\pi} = \left[\int_0^\infty s^{-1}\, J_1(s)J_0(s\rho)\, e^{-s|\mathrm{z}_i|}\, \mathrm{d}s\right]_+^-. \tag{22}$$

As for $\tilde{\varphi}_\perp$, once introduced (with minor abuse of notation) $\delta := +\sqrt{\rho^2+1-2\rho\, c_\psi}$, by leveraging Equation (20) (with $\mathrm{z}-\mathrm{z}'$ in place of z_i) and Equation (21) (with $\rho'=1$), it follows that:

$$\int_0^{2\pi} \frac{c_{\phi'}\,\mathrm{d}\phi'}{\sqrt{\delta^2+(\mathrm{z}-\mathrm{z}')^2}} = 2\pi\, c_\phi \int_0^\infty J_1(s)J_1(s\rho)\, e^{-s|\mathrm{z}-\mathrm{z}'|}\,\mathrm{d}s,$$

so that

$$\frac{\tilde{\varphi}_\perp}{2\pi} = c_\phi \int_{-\mathrm{L}}^{\mathrm{L}} \mathrm{d}\mathrm{z}' \int_0^\infty J_1(s)J_1(s\rho)\, e^{-s|\mathrm{z}-\mathrm{z}'|}\,\mathrm{d}s$$

and, upon integration over z', the sought expression for $\tilde{\varphi}_\perp$ finally reads:

$$\frac{\tilde{\varphi}_\perp}{2\pi} = \begin{cases} c_\phi \left[\int_0^\infty \frac{J_1(s)J_1(s\rho)}{s} e^{-s\mathrm{z}_i}\mathrm{d}s\right]_+^- & \text{for } \mathrm{z}\geq\mathrm{L} \\ c_\phi \int_0^\infty \frac{J_1(s)J_1(s\rho)}{s}\left(2-e^{s\mathrm{z}_-}-e^{-s\mathrm{z}_+}\right)\mathrm{d}s & \text{for } |\mathrm{z}|<\mathrm{L} \\ c_\phi \left[\int_0^\infty \frac{J_1(s)J_1(s\rho)}{s} e^{s\mathrm{z}_i}\mathrm{d}s\right]_-^+ & \text{for } \mathrm{z}\leq-\mathrm{L}. \end{cases} \tag{23}$$

For ease of presentation, we anticipate that, in light of Equation (23), at Step2 we proceeded by considering the following integrals involving products of Bessel functions:

$$\mathcal{I}^\lambda_{\alpha\beta}(a,b,c) := \int_0^\infty s^\lambda\, J_\alpha(s\,a)J_\beta(s\,b)\,e^{-s\,c}\,\mathrm{d}s,$$

with $\alpha,\beta,\lambda\in\mathbb{Z}$, such that: $\alpha+\beta+\lambda>-1$ if $c>0$; $\alpha+\beta+1>-\lambda>-1$ if $c=0$ and $a\neq b$; $\alpha+\beta+1>-\lambda>0$ if $c=0$ and $a=b$ (in order to recall relevant expressions from [SR5]). Moreover, we separately addressed the three cases $\mathrm{z}\geq\mathrm{L}$, $|\mathrm{z}|<\mathrm{L}$ and $\mathrm{z}\leq-\mathrm{L}$, for ease of derivation.

S3. Magnetic field and gradient: Solution details for Step2-3, case $\mathrm{z}\geq\mathrm{L}$

For $\mathrm{z}\geq\mathrm{L}$, $|\mathrm{z}_+|=\mathrm{z}_+$ and $|\mathrm{z}_-|=\mathrm{z}_-$ (relevant expressions from Table 4 are tacitly recalled). As for axial contributions, the following relations are derived from Equation (22) (also using $\partial_\rho J_0(s\rho)=-sJ_1(s\rho)$):

$$\tilde{\varphi}_\parallel = 2\pi\left[\mathcal{I}^{-1}_{10}(1,\rho,\mathrm{z}_i)\right]_+^- \tag{24}$$

$$-\partial_\rho\tilde{\varphi}_\parallel = 2\pi\left[\mathcal{I}^{0}_{11}(1,\rho,\mathrm{z}_i)\right]_+^- \tag{25}$$

$$-\partial_\mathrm{z}\tilde{\varphi}_\parallel = 2\pi\left[\mathcal{I}^{0}_{10}(1,\rho,\mathrm{z}_i)\right]_+^- \tag{26}$$

$$-\partial_\rho\partial_\mathrm{z}\tilde{\varphi}_\parallel = 2\pi\left[\mathcal{I}^{1}_{11}(1,\rho,\mathrm{z}_i)\right]_-^+ \tag{27}$$

$$-\partial_\mathrm{z}\partial_\mathrm{z}\tilde{\varphi}_\parallel = 2\pi\left[\mathcal{I}^{1}_{10}(1,\rho,\mathrm{z}_i)\right]_-^+. \tag{28}$$

By recalling ([SR5] Table3: ENS-4.2, ENS-4.7), Equation (25) and (26) can be recast in terms of $\mathcal{C}$ and Λ, thus leading to Equation (1) and (2), respectively. Similarly, by recalling ([SR5] Table3: ENS-4.4, ENS-4.8), Equation (27) and (28) respectively lead to Equation (8) and (9). For completeness, by using ([SR5] Table3: ENS-4.6) we also recast Equation (24) as follows:

$$\frac{\tilde{\varphi}_{\parallel}}{2} = \Big[\mathcal{C}\big(k_{ci}, 1, \varpi_i, \varpi_i - \mathrm{d}_i k_i^2\big)\Big]_{+}^{-} + 2\pi\,\mathrm{L}\,\mathcal{H}(1-\rho) + \mathrm{sign}(1-\rho)\,\Big[\mathrm{z}_i\,\Lambda(\sigma_i^2, k_i)\Big]_{+}^{-}, \tag{29}$$

with $\varpi_i := \mathrm{d}_i + (1-\rho^2)/\mathrm{d}_i$. Let us observe that $\tilde{\varphi}_{\parallel}$ is continuous, in particular across τ_1 (in Figure 1a).

As regards diametric contributions, the following relations are derived from Equation (23) (also using $\partial_\rho J_1(s\rho) = s J_0(s\rho) - \rho^{-1} J_1(s\rho)$):

$$\tilde{\varphi}_{\perp} = 2\pi\, c_\phi \Big[\mathcal{I}_{11}^{-1}(1,\rho,\mathrm{z}_i)\Big]_{+}^{-} \tag{30}$$

$$-\partial_\rho\tilde{\varphi}_{\perp} = 2\pi\, c_\phi \Big[\rho^{-1}\mathcal{I}_{11}^{-1}(1,\rho,\mathrm{z}_i) - \mathcal{I}_{10}^{0}(1,\rho,\mathrm{z}_i)\Big]_{+}^{-} \tag{31}$$

$$-\partial_\phi\tilde{\varphi}_{\perp} = 2\pi\, s_\phi \Big[\mathcal{I}_{11}^{-1}(1,\rho,\mathrm{z}_i)\Big]_{+}^{-} \tag{32}$$

$$-\partial_{\mathrm{z}}\tilde{\varphi}_{\perp} = 2\pi\, c_\phi \Big[\mathcal{I}_{11}^{0}(1,\rho,\mathrm{z}_i)\Big]_{+}^{-} \tag{33}$$

$$-\partial_\phi\partial_\rho\tilde{\varphi}_{\perp} = 2\pi\, s_\phi \Big[\rho^{-1}\mathcal{I}_{11}^{-1}(1,\rho,\mathrm{z}_i) - \mathcal{I}_{10}^{0}(1,\rho,\mathrm{z}_i)\Big]_{-}^{+} \tag{34}$$

$$-\partial_\phi\partial_\phi\tilde{\varphi}_{\perp} = 2\pi\, c_\phi \Big[\mathcal{I}_{11}^{-1}(1,\rho,\mathrm{z}_i)\Big]_{+}^{-} \tag{35}$$

$$-\partial_\rho\partial_{\mathrm{z}}\tilde{\varphi}_{\perp} = 2\pi\, c_\phi \Big[\mathcal{I}_{10}^{1}(1,\rho,\mathrm{z}_i) - \rho^{-1}\mathcal{I}_{11}^{0}(1,\rho,\mathrm{z}_i)\Big]_{+}^{-} \tag{36}$$

$$-\partial_\phi\partial_{\mathrm{z}}\tilde{\varphi}_{\perp} = 2\pi\, s_\phi \Big[\mathcal{I}_{11}^{0}(1,\rho,\mathrm{z}_i)\Big]_{-}^{+} \tag{37}$$

$$-\partial_{\mathrm{z}}\partial_{\mathrm{z}}\tilde{\varphi}_{\perp} = 2\pi\, c_\phi \Big[\mathcal{I}_{11}^{1}(1,\rho,\mathrm{z}_i)\Big]_{-}^{+}. \tag{38}$$

By recalling ([SR5] Table3: ENS-4.2, ENS-4.7, ENS-4.9), Equation (31), (32) and (33) can be recast in terms of $\mathcal{C}$ and Λ, thus leading to Equation (3), (4) and (5), respectively. Similarly, by recalling ([SR5] Table3: ENS-4.2, ENS-4.4, ENS-4.7, ENS-4.8, ENS-4.9), Equation (34), (35), (36), (37) and (38) respectively lead to Equation (10), (11), (12), (13) and (14). For completeness, by using ([SR5] Table3: ENS-4.9) we also recast Equation (30) as follows:

$$\tilde{\varphi}_{\perp} = c_\phi\,\rho^{-1}\left(\Big[\frac{\mathrm{z}_i}{\mathrm{d}_i}\,\mathcal{C}\big(k_{ci}, 1, (1-\rho)^2, (1+\rho)^2\big)\Big]_{-}^{+} - (1-\rho^2)\,\mathrm{sign}(1-\rho)\,\Big[\Lambda(\sigma_i^2, k_i)\Big]_{-}^{+}\right). \tag{39}$$

Let us observe that $\tilde{\varphi}_{\perp}$ is continuous, in particular across τ_1, and it can can be robustly computed even for $\rho\to 0$, since ([SR4] 6.611-1) $\mathcal{I}_{11}^{-1}(1,\rho\to 0,\mathrm{z}_i) = (\rho/2)(\sqrt{\mathrm{z}_i^2+1} - \mathrm{z}_i)/\sqrt{\mathrm{z}_i^2+1} + O(\rho^3) = O(\rho)$.

Finally, even f_2 (defined in Table 4) can be robustly computed for $\rho\to 0$, since the underlying term is given by $\rho^{-2}\,\gamma(\rho,\mathrm{z}_i)$, with $\gamma(\rho,\mathrm{z}_i) := 2\,\mathcal{I}_{11}^{-1}(1,\rho,\mathrm{z}_i) - \rho\,\mathcal{I}_{10}^{0}(1,\rho,\mathrm{z}_i)$, so that ([SR4] 6.621-4) $\gamma(\rho\to 0,\mathrm{z}_i) = \rho^3(3\,\mathrm{z}_i/8)(1+\mathrm{z}_i^2)^{-5/2} + O(\rho^5)$, whence $f_2 = O(\rho)$.

S4. Magnetic field and gradient: Solution details for Step2-3, case $|\mathrm{z}| < \mathrm{L}$

For $|\mathrm{z}|<\mathrm{L}$, $|\mathrm{z}_+|=\mathrm{z}_+$ and $|\mathrm{z}_-|=-\mathrm{z}_-$ (relevant expressions from Table 4 are tacitly recalled). As for axial contributions, the following relations are derived from Equation (22) (also using $\partial_\rho J_0(s\rho)=-sJ_1(s\rho)$):

$$\tilde{\varphi}_\parallel = 2\pi \Big(\mathcal{I}_{10}^{-1}(1,\rho,-\mathrm{z}_-) - \mathcal{I}_{10}^{-1}(1,\rho,\mathrm{z}_+)\Big) \tag{40}$$

$$-\partial_\rho\tilde{\varphi}_\parallel = 2\pi \Big(\mathcal{I}_{11}^{0}(1,\rho,-\mathrm{z}_-) - \mathcal{I}_{11}^{0}(1,\rho,\mathrm{z}_+)\Big) \tag{41}$$

$$-\partial_\mathrm{z}\tilde{\varphi}_\parallel = 2\pi \Big(-\mathcal{I}_{10}^{0}(1,\rho,-\mathrm{z}_-) - \mathcal{I}_{10}^{0}(1,\rho,\mathrm{z}_+)\Big) \tag{42}$$

$$-\partial_\rho\partial_\mathrm{z}\tilde{\varphi}_\parallel = 2\pi \Big(\mathcal{I}_{11}^{1}(1,\rho,-\mathrm{z}_-) + \mathcal{I}_{11}^{1}(1,\rho,\mathrm{z}_+)\Big) \tag{43}$$

$$-\partial_\mathrm{z}\partial_\mathrm{z}\tilde{\varphi}_\parallel = 2\pi \Big(-\mathcal{I}_{10}^{1}(1,\rho,-\mathrm{z}_-) + \mathcal{I}_{10}^{1}(1,\rho,\mathrm{z}_+)\Big). \tag{44}$$

By recalling ([SR5] Table3: ENS-4.2, ENS-4.7), Equation (41) and (42) can be recast in terms of $\mathcal{C}$ and Λ, thus leading to Equation (1) and (2), respectively. Similarly, by recalling ([SR5] Table3: ENS-4.4, ENS-4.8), Equation (43) and (44) respectively lead to Equation (8) and (9). For completeness, by using ([SR5] Table3: ENS-4.6) we also recast Equation (40) as follows:

$$\frac{\tilde{\varphi}_\parallel}{2} = \Big[\mathcal{C}\big(k_{ci},1,\varpi_i,\varpi_i-\mathrm{d}_i k_i^2\big)\Big]_+^- + 2\pi\,\mathrm{z}\,\mathcal{H}(1-\rho)\; - \;\mathrm{sign}(1-\rho)\;\Big(\mathrm{z}_-\,\Lambda(\sigma_-^2,k_-) + \mathrm{z}_+\,\Lambda(\sigma_+^2,k_+)\Big), \tag{45}$$

with $\varpi_i := \mathrm{d}_i + (1-\rho^2)/\mathrm{d}_i$. Let us observe that $\tilde{\varphi}_\parallel$ is continuous, in particular across S_l (in Figure 1a).

As regards diametric contributions, the following relations are derived from Equation (23) (also using $\partial_\rho J_1(s\rho)=sJ_0(s\rho)-\rho^{-1}J_1(s\rho)$):

$$\tilde{\varphi}_\perp = 2\pi\, c_\phi \Big(2\,\mathcal{I}_{11}^{-1}(1,\rho,0) - \mathcal{I}_{11}^{-1}(1,\rho,-\mathrm{z}_-) - \mathcal{I}_{11}^{-1}(1,\rho,\mathrm{z}_+)\Big) \tag{46}$$

$$\begin{aligned}-\partial_\rho\tilde{\varphi}_\perp = 2\pi\, c_\phi \Big(&-2\,\mathcal{I}_{10}^{0}(1,\rho,0) + \mathcal{I}_{10}^{0}(1,\rho,-\mathrm{z}_-) + \mathcal{I}_{10}^{0}(1,\rho,\mathrm{z}_+) + \\ &-\rho^{-1}\left(-2\,\mathcal{I}_{11}^{-1}(1,\rho,0) + \mathcal{I}_{11}^{-1}(1,\rho,-\mathrm{z}_-) + \mathcal{I}_{11}^{-1}(1,\rho,\mathrm{z}_+)\right)\Big)\end{aligned} \tag{47}$$

$$-\partial_\phi\tilde{\varphi}_\perp = 2\pi\, s_\phi \Big(2\,\mathcal{I}_{11}^{-1}(1,\rho,0) - \mathcal{I}_{11}^{-1}(1,\rho,-\mathrm{z}_-) - \mathcal{I}_{11}^{-1}(1,\rho,\mathrm{z}_+)\Big) \tag{48}$$

$$-\partial_\mathrm{z}\tilde{\varphi}_\perp = 2\pi\, c_\phi \Big(\mathcal{I}_{11}^{0}(1,\rho,-\mathrm{z}_-) - \mathcal{I}_{11}^{0}(1,\rho,\mathrm{z}_+)\Big) \tag{49}$$

$$\begin{aligned}-\partial_\phi\partial_\rho\tilde{\varphi}_\perp = 2\pi\, s_\phi \Big(&2\,\mathcal{I}_{10}^{0}(1,\rho,0) - \mathcal{I}_{10}^{0}(1,\rho,-\mathrm{z}_-) - \mathcal{I}_{10}^{0}(1,\rho,\mathrm{z}_+) + \\ &-\rho^{-1}\left(2\,\mathcal{I}_{11}^{-1}(1,\rho,0) - \mathcal{I}_{11}^{-1}(1,\rho,-\mathrm{z}_-) - \mathcal{I}_{11}^{-1}(1,\rho,\mathrm{z}_+)\right)\Big)\end{aligned} \tag{50}$$

$$-\partial_\phi\partial_\phi\tilde{\varphi}_\perp = 2\pi\, c_\phi \Big(2\,\mathcal{I}_{11}^{-1}(1,\rho,0) - \mathcal{I}_{11}^{-1}(1,\rho,-\mathrm{z}_-) - \mathcal{I}_{11}^{-1}(1,\rho,\mathrm{z}_+)\Big) \tag{51}$$

$$-\partial_\rho\partial_\mathrm{z}\tilde{\varphi}_\perp = 2\pi\, c_\phi \Big(\mathcal{I}_{10}^{1}(1,\rho,-\mathrm{z}_-) - \mathcal{I}_{10}^{1}(1,\rho,\mathrm{z}_+) - \rho^{-1}\left(\mathcal{I}_{11}^{0}(1,\rho,-\mathrm{z}_-) - \mathcal{I}_{11}^{0}(1,\rho,\mathrm{z}_+)\right)\Big) \tag{52}$$

$$-\partial_\phi\partial_\mathrm{z}\tilde{\varphi}_\perp = 2\pi\, s_\phi \Big(\mathcal{I}_{11}^{0}(1,\rho,\mathrm{z}_+) - \mathcal{I}_{11}^{0}(1,\rho,-\mathrm{z}_-)\Big) \tag{53}$$

$$-\partial_\mathrm{z}\partial_\mathrm{z}\tilde{\varphi}_\perp = 2\pi\, c_\phi \Big(\mathcal{I}_{11}^{1}(1,\rho,-\mathrm{z}_-) + \mathcal{I}_{11}^{1}(1,\rho,\mathrm{z}_+)\Big). \tag{54}$$

By recalling ([SR5] Table2 - where $\mathcal{I}^0_{10}(1,1,0)$, although not formally included in the given definition of $\mathcal{I}^\lambda_{\alpha\beta}$, is correctly reported, see ([SR4] 6.512-3)) and ([SR5] Table3: ENS-4.2, ENS-4.7, ENS-4.9), Equation (47), (48) and (49) can be recast in terms of $\mathcal{C}$ and Λ, thus leading to Equation (3), (4) and (5), respectively. Similarly, by recalling ([SR5] Table3: ENS-4.2, ENS-4.4, ENS-4.7, ENS-4.8, ENS-4.9), Equation (50), (51), (52), (53) and (54) respectively lead to Equation (10), (11), (12), (13) and (14). For completeness, by using ([SR5] Table2, Table3: ENS-4.9) we also recast Equation (46) as follows:

$$\tilde{\varphi}_\perp = c_\phi\,\rho^{-1}\left(\left[\frac{\mathrm{z}_i}{\mathrm{d}_i}\,\mathcal{C}\big(k_{ci},1,(1-\rho)^2,(1+\rho)^2\big)\right]_-^+ - (1-\rho^2)\,\mathrm{sign}(1-\rho)\,\Big(\Lambda(\sigma_+^2,k_+)+\Lambda(\sigma_-^2,k_-)\Big)\right). \tag{55}$$

Let us observe that $\tilde{\varphi}_\perp$ is continuous, in particular across S_l, and it can can be robustly computed even for $\rho\to 0$, since ([SR4] 6.611-1, 6.561-14) $2\,\mathcal{I}^{-1}_{11}(1,\rho\to 0,0)-\mathcal{I}^{-1}_{11}(1,\rho\to 0,-\mathrm{z}_-)-\mathcal{I}^{-1}_{11}(1,\rho\to 0,\mathrm{z}_+) = (\rho/2)\Big(2-(\sqrt{\mathrm{z}_-^2+1}+\mathrm{z}_-)/\sqrt{\mathrm{z}_-^2+1}-(\sqrt{\mathrm{z}_+^2+1}-\mathrm{z}_+)/\sqrt{\mathrm{z}_+^2+1}\Big)+O(\rho^3)=O(\rho)$.

Finally, even f_2 (defined in Table 4) can be robustly computed for $\rho\to 0$, since the underlying terms are given by $\rho^{-2}\,\gamma_0(\rho)$ and $\rho^{-2}\,\gamma_\pm(\rho,\pm\mathrm{z}_\pm)$, with $\gamma_0(\rho):=2\,\mathcal{I}^{-1}_{11}(1,\rho,0)-\rho\,\mathcal{I}^0_{10}(1,\rho,0)$ and $\gamma_\pm(\rho,\pm\mathrm{z}_\pm):=2\,\mathcal{I}^{-1}_{11}(1,\rho,\pm\mathrm{z}_\pm)-\rho\,\mathcal{I}^0_{10}(1,\rho,\pm\mathrm{z}_\pm)$, so that ([SR4] 6.512-3, 6.621-4) $\gamma_0(\rho<1)=\rho\int_0^\infty J_1(s)J_2(\rho s)\mathrm{d}s\equiv 0$ and $\gamma_\pm(\rho\to 0,\pm\mathrm{z}_\pm)=\rho^3(\pm 3\,\mathrm{z}_\pm/8)(1+\mathrm{z}_\pm^2)^{-5/2}+O(\rho^5)$, whence $f_2=O(\rho)$.

S5. Magnetic field and gradient: Solution details for Step2-3, case $\mathrm{z}\le -\mathrm{L}$

For $\mathrm{z}\le-\mathrm{L}$, $|\mathrm{z}_+|=-\mathrm{z}_+$ and $|\mathrm{z}_-|=-\mathrm{z}_-$ (relevant expressions from Table 4 are tacitly recalled). As for axial contributions, the following relations are derived from Equation (22) (also using $\partial_\rho J_0(s\rho)=-sJ_1(s\rho)$):

$$\tilde{\varphi}_\parallel = 2\pi\left[\mathcal{I}^{-1}_{10}(1,\rho,-\mathrm{z}_i)\right]_+^- \tag{56}$$
$$-\partial_\rho\tilde{\varphi}_\parallel = 2\pi\left[\mathcal{I}^{0}_{11}(1,\rho,-\mathrm{z}_i)\right]_+^- \tag{57}$$
$$-\partial_\mathrm{z}\tilde{\varphi}_\parallel = 2\pi\left[\mathcal{I}^{0}_{10}(1,\rho,-\mathrm{z}_i)\right]_-^+ \tag{58}$$
$$-\partial_\rho\partial_\mathrm{z}\tilde{\varphi}_\parallel = 2\pi\left[\mathcal{I}^{1}_{11}(1,\rho,-\mathrm{z}_i)\right]_+^- \tag{59}$$
$$-\partial_\mathrm{z}\partial_\mathrm{z}\tilde{\varphi}_\parallel = 2\pi\left[\mathcal{I}^{1}_{10}(1,\rho,-\mathrm{z}_i)\right]_-^+ . \tag{60}$$

By recalling ([SR5] Table3: ENS-4.2, ENS-4.7), Equation (57) and (58) can be recast in terms of $\mathcal{C}$ and Λ, thus leading to Equation (1) and (2), respectively. Similarly, by recalling ([SR5] Table3: ENS-4.4, ENS-4.8), Equation (59) and (60) respectively lead to Equation (8) and (9). For completeness, by using ([SR5] Table3: ENS-4.6) we also recast Equation (56) as follows:

$$\frac{\tilde{\varphi}_\parallel}{2} = \left[\mathcal{C}\big(k_{ci},1,\varpi_i,\varpi_i-\mathrm{d}_i k_i^2\big)\right]_+^- - 2\pi\,\mathrm{L}\,\mathcal{H}(1-\rho) - \mathrm{sign}(1-\rho)\left[\mathrm{z}_i\,\Lambda(\sigma_i^2,k_i)\right]_+^-, \tag{61}$$

with $\varpi_i:=\mathrm{d}_i+(1-\rho^2)/\mathrm{d}_i$. Let us observe that $\tilde{\varphi}_\parallel$ is continuous, in particular across τ_l (in Figure 1a).

As regards diametric contributions, the following relations are derived from Equation (23) (also using $\partial_\rho J_1(s\rho)=sJ_0(s\rho)-\rho^{-1}J_1(s\rho)$):

$$\tilde{\varphi}_\perp = 2\pi c_\phi \left[\mathcal{I}_{11}^{-1}(1,\rho,-\mathrm{z}_i)\right]_-^+ \tag{62}$$

$$-\partial_\rho\tilde{\varphi}_\perp = 2\pi c_\phi \left[\rho^{-1}\mathcal{I}_{11}^{-1}(1,\rho,-\mathrm{z}_i) - \mathcal{I}_{10}^{0}(1,\rho,-\mathrm{z}_i)\right]_-^+ \tag{63}$$

$$-\partial_\phi\tilde{\varphi}_\perp = 2\pi s_\phi \left[\mathcal{I}_{11}^{-1}(1,\rho,-\mathrm{z}_i)\right]_-^+ \tag{64}$$

$$-\partial_\mathrm{z}\tilde{\varphi}_\perp = 2\pi c_\phi \left[\mathcal{I}_{11}^{0}(1,\rho,-\mathrm{z}_i)\right]_+^- \tag{65}$$

$$-\partial_\phi\partial_\rho\tilde{\varphi}_\perp = 2\pi s_\phi \left[\rho^{-1}\mathcal{I}_{11}^{-1}(1,\rho,-\mathrm{z}_i) - \mathcal{I}_{10}^{0}(1,\rho,-\mathrm{z}_i)\right]_+^- \tag{66}$$

$$-\partial_\phi\partial_\phi\tilde{\varphi}_\perp = 2\pi c_\phi \left[\mathcal{I}_{11}^{-1}(1,\rho,-\mathrm{z}_i)\right]_-^+ \tag{67}$$

$$-\partial_\rho\partial_\mathrm{z}\tilde{\varphi}_\perp = 2\pi c_\phi \left[\mathcal{I}_{10}^{1}(1,\rho,-\mathrm{z}_i) - \rho^{-1}\mathcal{I}_{11}^{0}(1,\rho,-\mathrm{z}_i)\right]_+^- \tag{68}$$

$$-\partial_\phi\partial_\mathrm{z}\tilde{\varphi}_\perp = 2\pi s_\phi \left[\mathcal{I}_{11}^{0}(1,\rho,-\mathrm{z}_i)\right]_-^+ \tag{69}$$

$$-\partial_\mathrm{z}\partial_\mathrm{z}\tilde{\varphi}_\perp = 2\pi c_\phi \left[\mathcal{I}_{11}^{1}(1,\rho,-\mathrm{z}_i)\right]_+^- . \tag{70}$$

By recalling ([SR5] Table3: ENS-4.2, ENS-4.7, ENS-4.9), Equation (63), (64) and (65) can be recast in terms of $\mathcal{C}$ and Λ, thus leading to Equation (3), (4) and (5), respectively. Similarly, by recalling ([SR5] Table3: ENS-4.2, ENS-4.4, ENS-4.7, ENS-4.8, ENS-4.9), Equation (66), (67), (68), (69) and (70) respectively lead to Equation (10), (11), (12), (13) and (14). For completeness, by using ([SR5] Table3: ENS-4.9) we also recast Equation (62) as follows:

$$\tilde{\varphi}_\perp = c_\phi\,\rho^{-1}\left(\left[\frac{\mathrm{z}_i}{\mathrm{d}_i}\,\mathcal{C}\big(k_{ci},1,(1-\rho)^2,(1+\rho)^2\big)\right]_-^+ + (1-\rho^2)\ \mathrm{sign}(1-\rho)\left[\Lambda(\sigma_i^2,k_i)\right]_-^+\right). \tag{71}$$

Let us observe that $\tilde{\varphi}_\perp$ is continuous, in particular across τ_1, and it can can be robustly computed even for $\rho\to 0$, since ([SR4] 6.611-1) $\mathcal{I}_{11}^{-1}(1,\rho\to 0,-\mathrm{z}_i) = (\rho/2)(\sqrt{\mathrm{z}_i^2+1}+\mathrm{z}_i)/\sqrt{\mathrm{z}_i^2+1} + O(\rho^3) = O(\rho)$.

Finally, even f_2 (defined in Table 4) can be robustly computed for $\rho\to 0$, since the underlying term is given by $\rho^{-2}\,\gamma(\rho,\mathrm{z}_i)$, with $\gamma(\rho,\mathrm{z}_i) := 2\,\mathcal{I}_{11}^{-1}(1,\rho,-\mathrm{z}_i) - \rho\,\mathcal{I}_{10}^{0}(1,\rho,-\mathrm{z}_i)$, so that ([SR4] 6.621-4) $\gamma(\rho\to 0,\mathrm{z}_i) = \rho^3(-3\,\mathrm{z}_i/8)(1+\mathrm{z}_i^2)^{-5/2} + O(\rho^5)$, whence $f_2 = O(\rho)$.

S6. Magnetic field and gradient: Solution details for Step4

With reference to Table 1 and 2, cylindrical components of both magnetic field and gradient are defined for $\rho > 0$, namely for off-axis $\mathbf{P}$ points, for ϕ (whence $\hat{\mathbf{e}}_\rho$ and $\hat{\mathbf{e}}_\phi$) to be defined. Differently, the corresponding complete solutions (namely Equation (6) and (15)) seamlessly hold in the whole computational domain. As for $\mathbf{H}$, Equation (6) circumvents the indeterminacy of ϕ for points $\mathbf{P}$ on the cylinder axis thanks to the fact that $\mathbf{u} = \rho\,\mathrm{M}_\perp(c_{2\phi}\,\hat{\mathbf{e}}_\mathrm{x} + s_{2\phi}\,\hat{\mathbf{e}}_\mathrm{y})$ and $\mathbf{v} = -\rho\,(\mathrm{M}_\parallel\,c_\phi\,\hat{\mathbf{e}}_\mathrm{x} + \mathrm{M}_\parallel\,s_\phi\,\hat{\mathbf{e}}_\mathrm{y} + \mathrm{M}_\perp\,c_\phi\,\hat{\mathbf{e}}_\mathrm{z})$, with both $\mathbf{u}$ and $\mathbf{v}$ vanishing for $\rho = 0$. More in detail, for $\rho > 0$, the above expression for $\mathbf{u}$ is recast in Table 4 as a reflection across the plane through the origin $\mathbf{O}$ with unit normal $\boldsymbol{\nu}$, and the definition of $\boldsymbol{\nu}$ consistently provides a continuous extension of $\mathbf{u}$ at $\rho = 0$. As for grad$\,(\mathbf{H})$, Equation (15) circumvents the indeterminacy of ϕ for points $\mathbf{P}$ on the cylinder axis thanks to the continuous extension provided through the definition of $\tilde{J}_\parallel$, $\tilde{J}_\perp$, $\tilde{c}$ and $\tilde{s}$ in Table 4. Let us remark that, in numerical implementations, the conditions $\rho > 0$ and $\rho = 0$ should be replaced by $\rho > \epsilon$ and $\rho \leq \epsilon$, respectively, where $\epsilon \ll 1$ is a chosen threshold.

S7. Magnetic scalar potential: Solution

The resulting expression of the magnetic scalar potential reads:

$$\varphi = \Big((\mathbf{p}\cdot\hat{\mathbf{e}}_\perp)\,(f_1+\rho\, f_2)\,\mathrm{M}_\perp - (\breve{f}_0+2\breve{f})\,\mathrm{M}_\parallel \Big)\,\bar{\mathrm{R}}/\,\pi, \tag{72}$$

where

$$\breve{f}(\rho,\mathrm{z};\mathrm{L}) := \frac{1}{4}\left(\left[\frac{1}{\mathrm{d}_i}\,\mathcal{C}\big(k_{ci},1,2\,(1+\rho)+\mathrm{z}_i^2,2\,(1-\rho)+\mathrm{z}_i^2\big) \right]_-^+ + \breve{f}_\Lambda \right)$$

$$\begin{cases} \breve{f}_\Lambda(\rho,\mathrm{z};\mathrm{L}) := \mathrm{sign}(1-\rho)\,\big[\mathrm{z}_i\,\Lambda(\sigma_i^2,k_i)\big]_-^+, & \breve{f}_0(\rho,\mathrm{z};\mathrm{L}) := -\pi\,\mathcal{H}(1-\rho)\ \mathrm{L} \quad \text{for } \mathrm{z}\geq \mathrm{L} \\ \breve{f}_\Lambda(\rho,\mathrm{z};\mathrm{L}) := \mathrm{sign}(1-\rho)\,\big(\mathrm{z}_+\,\Lambda(\sigma_+^2,k_+) + \mathrm{z}_-\,\Lambda(\sigma_-^2,k_-)\big), & \breve{f}_0(\rho,\mathrm{z};\mathrm{L}) := -\pi\,\mathcal{H}(1-\rho)\ \mathrm{z} \quad \text{for } |\mathrm{z}|<\mathrm{L} \\ \breve{f}_\Lambda(\rho,\mathrm{z};\mathrm{L}) := \mathrm{sign}(1-\rho)\,[\mathrm{z}_i\,\Lambda(\sigma_i^2,k_i)]_+^-, & \breve{f}_0(\rho,\mathrm{z};\mathrm{L}) := +\pi\,\mathcal{H}(1-\rho)\ \mathrm{L} \quad \text{for } \mathrm{z}\leq -\mathrm{L} \end{cases}$$

and the remaining terms are recalled from Table 4. More in detail, from Equation (29), (45) and (61), it follows that $\tilde{\varphi}_\parallel = -4\,(\breve{f}_0 + 2\breve{f})$. Furthermore, from Equation (39), (55) and (71), it follows that $\tilde{\varphi}_\perp = 4\,(c_\phi\,\rho)\,(f_1+\rho f_2) = 4\,(\mathbf{p}\cdot\hat{\mathbf{e}}_\perp)\,(f_1+\rho f_2)$, where the latter equality permits to circumvents the indeterminacy of ϕ for points $\mathbf{P}$ on the cylinder axis. Equation (72) is then obtained by recalling that $\varphi = \big(\mathrm{M}_\parallel\,\tilde{\varphi}_\parallel + \mathrm{M}_\perp\tilde{\varphi}_\perp\big)\,\bar{\mathrm{R}}/(4\pi)$. As anticipated in Section S3, S4, S5, Supporting Information, φ is continuous in the whole computational domain (as well as across the magnet surface).

S8. Magnetic force and torque: Solution details

Upon non-dimensionalization, $\mathrm{R}_2 := \bar{\mathrm{R}}_2/\bar{\mathrm{R}}_1$, $\mathrm{L}_1 := \bar{\mathrm{L}}_1/\bar{\mathrm{R}}_1$, $\mathrm{L}_2 := \bar{\mathrm{L}}_2/\bar{\mathrm{R}}_1$, $\mathrm{d} := \bar{\mathrm{d}}/\bar{\mathrm{R}}_1$ (and $\mathrm{R}_1 := \bar{\mathrm{R}}_1/\bar{\mathrm{R}}_1 = 1$). By using the results in Table 1 and 2, integration about the axial direction (namely the ϕ-sweep of the underlying volume integrals) provides:

$$\mathbf{f}_\parallel^{1\to2} = 2\mu_0\,\bar{\mathrm{R}}_1^2\ F_5\,(\mathbf{M}_{\parallel1}\cdot\mathbf{M}_{\parallel2})\,\hat{\mathbf{e}}^{1\to2} \tag{73}$$

$$\mathbf{f}_\perp^{1\to2} = -\mu_0\,\bar{\mathrm{R}}_1^2\ F_5\,(\mathbf{M}_{\perp1}\cdot\mathbf{M}_{\perp2})\,\hat{\mathbf{e}}^{1\to2} \tag{74}$$

$$\mathbf{t}_\perp^{1\to2} = 2\mu_0\,\bar{\mathrm{R}}_1^3\ F_1\,(\mathbf{M}_{\perp1}\times\mathbf{M}_{\perp2}), \tag{75}$$

with

$$F_1 := \int_{\rho=0}^{\mathrm{R}_2}\int_{\mathrm{z}=\mathrm{d}-\mathrm{L}_2}^{\mathrm{d}+\mathrm{L}_2} f_1(\rho,\mathrm{z};\mathrm{L}_1)\,\rho\,\mathrm{d}\rho\ \mathrm{dz} \qquad \text{and} \qquad F_5 := \int_{\rho=0}^{\mathrm{R}_2}\int_{\mathrm{z}=\mathrm{d}-\mathrm{L}_2}^{\mathrm{d}+\mathrm{L}_2} f_5(\rho,\mathrm{z};\mathrm{L}_1)\,\rho\,\mathrm{d}\rho\ \mathrm{dz}.$$

Based on the assumption that magnet C2 resides in the $\mathrm{z} > \mathrm{L}_1$ half-space (which does not affect the generality), from relevant expressions in Section S3, Supporting Information, one straightforwardly obtains $f_1 = (\pi/4)\,[\mathcal{I}_{10}^0(1,\rho,\mathrm{z}_i)]_+^-$ and $f_5 = (\pi/2)\,[\mathcal{I}_{10}^1(1,\rho,\mathrm{z}_i)]_-^+$, so that direct integration (first in ρ, then in z) leads to:

$$\begin{aligned} \frac{-4F_1}{\pi\mathrm{R}_2} &= \mathcal{I}_{11}^{-2}(1,\mathrm{R}_2,\mathrm{d}-\mathrm{L}_1+\mathrm{L}_2) + \mathcal{I}_{11}^{-2}(1,\mathrm{R}_2,\mathrm{d}+\mathrm{L}_1-\mathrm{L}_2) - \mathcal{I}_{11}^{-2}(1,\mathrm{R}_2,\mathrm{d}+\mathrm{L}_1+\mathrm{L}_2) - \mathcal{I}_{11}^{-2}(1,\mathrm{R}_2,\mathrm{d}-\mathrm{L}_1-\mathrm{L}_2) \\ \frac{2F_5}{\pi\mathrm{R}_2} &= \mathcal{I}_{11}^{-1}(1,\mathrm{R}_2,\mathrm{d}-\mathrm{L}_1+\mathrm{L}_2) + \mathcal{I}_{11}^{-1}(1,\mathrm{R}_2,\mathrm{d}+\mathrm{L}_1-\mathrm{L}_2) - \mathcal{I}_{11}^{-1}(1,\mathrm{R}_2,\mathrm{d}+\mathrm{L}_1+\mathrm{L}_2) - \mathcal{I}_{11}^{-1}(1,\mathrm{R}_2,\mathrm{d}-\mathrm{L}_1-\mathrm{L}_2)\,. \end{aligned}$$

Starting from the above expressions, the solutions in Table 3 can be obtained by recasting $\mathcal{I}_{11}^{-2}(1,\mathrm{R}_2,x)$ and $\mathcal{I}_{11}^{-1}(1,\mathrm{R}_2,x)$ in terms of $\mathcal{C}$, yet some attention must be paid to seamlessly encompass the cases $x>0$ and $x=0$, as sketched below.

First, by assuming $x>0$, we used ([SR5] Table4) to recast $\mathcal{I}_{11}^{-2}(1,\mathrm{R}_2,x)$ and $\mathcal{I}_{11}^{-1}(1,\mathrm{R}_2,x)$ in terms of $\mathcal{C}$, so as to finally map Equation (73), (74) and (75) onto Equation (16), (17), and (18), respectively. To the purpose, and with reference to Table 5, we introduced, in particular, $f_6(\mathrm{R}_2,x)$, $f_7(\mathrm{R}_2,x)$ and $f_8(\mathrm{R}_2,x)$, based on which we defined $\eta(\mathrm{R}_2,x) := x\,\tilde{\eta}(\mathrm{R}_2,x)$ and $\zeta(\mathrm{R}_2,x) := \tilde{\zeta}(\mathrm{R}_2,x)$, with $\tilde{\eta}(\mathrm{R}_2,x) := (f_6-f_7)/\ell$ and $\tilde{\zeta}(\mathrm{R}_2,x) := ((2(1+\mathrm{R}_2^2)-x^2)f_6+f_8)/\ell$. Contextually, we introduced ξ in order to seamlessly deal with all the three cases $\mathrm{R}_2 \lesseqgtr 1$. We then addressed the case $x=0$, by considering the corresponding limit expressions, namely $f_6(\mathrm{R}_2,0) = -\xi D(\xi)$, $f_7(\mathrm{R}_2,0) = \xi D(\xi)$ and $f_8(\mathrm{R}_2,0) = 4\mathrm{R}_2 K(\xi)$, with $D(\xi) := \mathcal{C}\left(\sqrt{1-\xi^2},1,0,1\right)$, whence $\tilde{\eta}(\mathrm{R}_2,0) = -2\,\xi D(\xi)/\ell(\xi)$ and $\tilde{\zeta}(\mathrm{R}_2,0) = 2\,(2\mathrm{R}_2\,E(\xi) - \xi\,(1+\mathrm{R}_2^2 - 2\mathrm{R}_2\,\xi)\,D(\xi))\,/\ell(\xi)$, with $\ell(\xi)=1$ for $\mathrm{R}_2 \le 1$ and $\ell(\xi)=\mathrm{R}_2$ for $\mathrm{R}_2>1$. Both $\tilde{\eta}(\mathrm{R}_2,0)$ and $\tilde{\zeta}(\mathrm{R}_2,0)$ (whose expression can be alternatively obtained by initially recasting $\mathcal{I}_{11}^{-2}(1,\mathrm{R}_2,0)$ and $\mathcal{I}_{11}^{-1}(1,\mathrm{R}_2,0)$ in terms of $\mathcal{C}$ through ([SR5] Table2)) can be straightforwardly computed for $0<\xi<1$, thus allowing to immediately extend the above definitions of η and ζ to $(\mathrm{R}_2,0)$, with the only exception of $\mathrm{R}_2=1$ (corresponding to $\xi=1$). By observing that $\eta(\mathrm{R}_2,0)=0$ for $0<\xi<1$, we extended η by continuity, by defining $\eta(1,0):=0$, thus circumventing the fact that $\tilde{\eta}(1,0)$ is not defined since $D(1)$ diverges (regardless of the fact that the corresponding expression, namely $\mathcal{C}(0,1,0,1)$, is not formally included in the given definition of $\mathcal{C}$, namely Equation (19), for which the first two arguments are assumed to be different from zero). Finally, although $\tilde{\zeta}(\mathrm{R}_2,0)$ is defined even for $\mathrm{R}_2=1$, and in particular $\tilde{\zeta}(1,0)=4$, we introduced a continuous extension for ζ as well, by defining $\zeta(1,0):=4$. This is due to the fact that $\tilde{\zeta}(\mathrm{R}_2,0) = 2\,\mathcal{C}\left(\sqrt{1-\xi^2},1,2\mathrm{R}_2,2\mathrm{R}_2\,(1-\xi^2)-\xi\,(1+\mathrm{R}_2^2-2\mathrm{R}_2\,\xi)\right)/\ell(\xi)$, so that $\tilde{\zeta}(1,0)=4\,\mathcal{C}(0,1,1,0)$ is not formally included in the given definition of $\mathcal{C}$, for the same reason recalled above. Let us remark that, in numerical implementations, the condition $(\mathrm{R}_2,x)\neq(1,0)$ should be replaced by $|\mathrm{R}_2-1|\le\epsilon_\mathrm{R}$ and $x\le\epsilon_x$, where $\epsilon_\mathrm{R}\ll 1$ and $\epsilon_x\ll 1$ are chosen thresholds.

S9. References

[SR1] NIST Digital Library of Mathematical Functions (release 1.1.5 of 2022-03-15), http://dlmf.nist.gov, National Institute of Standards and Technology, **2022**.

[SR2] M. Batista, *SoftwareX* **2019**, *10* 100245.

[SR3] P. F. Byrd, M. D. Friedman, *Handbook of elliptic integrals for engineers and physicists*, volume 67, Springer, **2013**.

[SR4] I. S. Gradshteyn, I. M. Ryzhik, *Table of integrals, series, and products*, Academic Press, **2014**.

[SR5] E. Kausel, M. Irfan Baig, *Quarterly of applied mathematics* **2012**, *70*, 1 77.
